# Plant Polyphenols for Aging Health: Implication from Their Autophagy Modulating Properties in Age-Associated Diseases

**DOI:** 10.3390/ph14100982

**Published:** 2021-09-27

**Authors:** James Michael Brimson, Mani Iyer Prasanth, Dicson Sheeja Malar, Premrutai Thitilertdecha, Atul Kabra, Tewin Tencomnao, Anchalee Prasansuklab

**Affiliations:** 1 Natural Products for Neuroprotection and Anti-Ageing Research Unit, Chulalongkorn University, Bangkok 10330, Thailand; James.b@chula.ac.th (J.M.B.); prasanth.m.iyer@gmail.com (M.I.P.); sheeja.malar@gmail.com (D.S.M.); 2 Department of Clinical Chemistry, Faculty of Allied Health Sciences, Chulalongkorn University, Bangkok 10330, Thailand; 3 Siriraj Research Group in Immunobiology and Therapeutic Sciences, Faculty of Medicine Siriraj Hospital, Mahidol University, Bangkok 10330, Thailand; premrutai@gmail.com; 4 Department of Pharmacology, University Institute of Pharma Sciences, Chandigarh University, Sahibzad Ajit Singh Nagar 140413, Punjab, India; atul.kbr@gmail.com; 5 College of Public Health Sciences, Chulalongkorn University, Bangkok 10330, Thailand

**Keywords:** autophagosome, cancer, diabetes, neurodegenerative disease, natural products, resveratrol, caffeic acid, curcumin, epigallocatechin gallate, luteolin

## Abstract

Polyphenols are a family of naturally occurring organic compounds, majorly present in fruits, vegetables, and cereals, characterised by multiple phenol units, including flavonoids, tannic acid, and ellagitannin. Some well-known polyphenols include resveratrol, quercetin, curcumin, epigallocatechin gallate, catechin, hesperetin, cyanidin, procyanidin, caffeic acid, and genistein. They can modulate different pathways inside the host, thereby inducing various health benefits. Autophagy is a conserved process that maintains cellular homeostasis by clearing the damaged cellular components and balancing cellular survival and overall health. Polyphenols could maintain autophagic equilibrium, thereby providing various health benefits in mediating neuroprotection and exhibiting anticancer and antidiabetic properties. They could limit brain damage by dismantling misfolded proteins and dysfunctional mitochondria, thereby activating autophagy and eliciting neuroprotection. An anticarcinogenic mechanism is stimulated by modulating canonical and non-canonical signalling pathways. Polyphenols could also decrease insulin resistance and inhibit loss of pancreatic islet β-cell mass and function from inducing antidiabetic activity. Polyphenols are usually included in the diet and may not cause significant side effects that could be effectively used to prevent and treat major diseases and ailments.

## 1. Introduction

### 1.1. Polyphenolic Compounds

**Figure 1:**
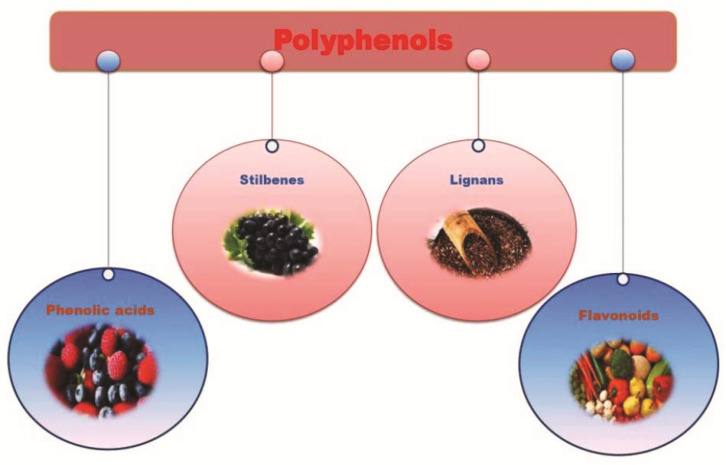
Polyphenol classification including phenolic acids, stilbenes, lignans and flavonoids.

Polyphenols are secondary plant metabolites and are technically termed from their chemical structure of repeating phenolic moieties (i.e., a phenyl ring encompasses a benzene ring substituted with at least one hydroxyl group) with/without other functional groups (e.g., esters and glycosides). Polyphenols can be identified by several phenol rings, structural elements connecting the rings together, and substituents attached to those rings [1]. They are commonly classified into four main groups, including phenolic acids, flavonoids, stilbenes, and lignans (Figure 1) [2]. Phenolic acids can be further identified as hydrobenzoic acids and hydroxycinnamic acids, and flavonoids, which are subsequently divided into flavonols, flavones, flavanones, isoflavones, anthocyanidins, and flavanols. Over several hundred polyphenols have been discovered in edible plants such as fruits, vegetables, nuts, and seeds. Polyphenolic compounds are usually in the form of glycone (i.e., O-glycoside, with a sugar group) and later converted into an aglycone form (i.e., a nonsugar component) upon oral ingestion through a hydroxylation reaction by β-glucosidase enzymes in the digestive system before absorption via passive diffusion of protein carriers. These aglycones are then conjugated into O-glucuronides or O-sulphates in the liver and transported into the bloodstream until urinary excretion [3].

**Figure 2:**
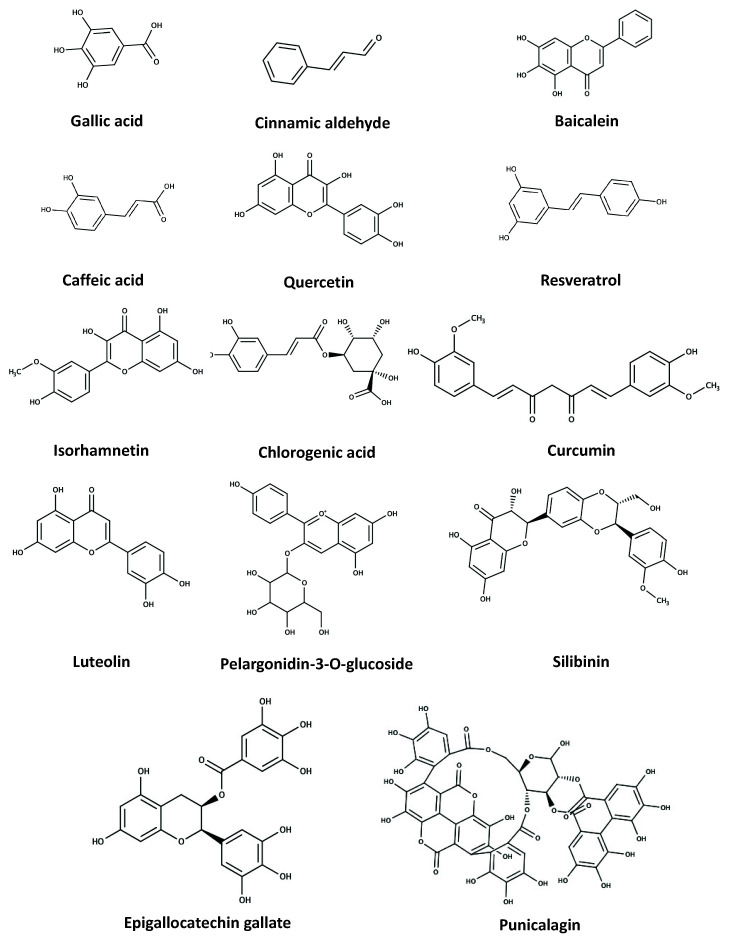
Structures of common polyphenolic compounds.

Interestingly, this individual polyphenol can generate more than one metabolite byproduct in the body, resulting in different active roles and interfering with signalling pathways for various biological activities [4]. In this review, compounds from various groups of polyphenols (Figure 2) including hydroxycinnamic acids (caffeic acid and curcumin), flavanols (quercetin), flavanones (hesperetin), isoflavones (genistein), anthocyanidins (cyanidin and procyanidin), flavanols (catechin and epigallocatechin gallate), and stilbenes (resveratrol) are focused on due to their feasibility in autophagy adapting properties and abundance in daily diets. Caffeic acid is the most abundant phenolic acid in fruits, with 75–100% of total hydroxycinnamic acid content, especially in the outer parts of ripe fruits [1]. It is available in kiwi, blueberries, cranberries, buckwheat grits, wheat kernels, and coffee [1,5,6,7,8]. For curcumin, it is a well-known active species in turmeric which is a spice broadly used in Southeast Asian countries’ cuisines and traditional medicines [9]. Quercetin is one of the most studied flavonols found in buckwheat, asparagus, citrus fruits, peaches, apples, green tea, curly kale, lovages, and dills [1,3,10]. Hesperetin can be obtained from grapefruit juice, oranges, and cheeses [11,12], while genistein’s best-known source is soy-based foods and beans, black beans, chickpeas, and peas [13,14]. Although cyanidin is considered as the most common anthocyanidin in foods and is accessible from blackberries, blackcurrants, and drinking yoghurts [1,15], there is more evidence for procyanidin’s presence in numerous fruits, vegetables, nuts, and grains such as apples, grapes, kiwi, bananas, mangoes, cherries, strawberries, tomatoes, pineapples, carrots, aubergines, potatoes, almonds, peanuts, and rice as well as chocolate and wine [16,17]. Regarding catechin, it can be found in various fruits such as grapes, apples, pears, cherries, and kiwi as well as dark chocolate, green tea, and fruit wines [18,19,20], while epigallocatechin gallate (an ester of epigallocatechin and gallic acid) is predominant in green tea, cocoa-based products, and cheese [12,21]. Unlike the other compounds, resveratrol is usually present in the diet in low quantities even though it can be found in many foods, particularly grapes, berries, peanuts, and wines [21,22].

### 1.2. Autophagy

Macroautophagy, otherwise known as autophagy, is a conserved catabolic mechanism, which degrades excess, unrequired, or damaged cellular components. Autophagy is under the control of several pathways and thus a target for multiple disease states, including bacterial infection [23], viral infection [24], neurodegenerative diseases [25], cancer [26], and diabetes [27]. Autophagy has been studied extensively in yeast (*Saccharomyces cerevisiae*) as a mechanism to stand starvation conditions [28], where the yeast cells would consume their cellular components, attempting to survive starvation, and this is the origin of the term autophagy (literally meaning self-eating). Furthermore, many genes in yeast that control autophagy have been identified and are denoted as autophagy-related genes (Atg) [29], many of these have mammalian homologs. Some of the main pathways controlling autophagy include the mechanistic target of rapamycin (mTOR) pathway and 5′ adenosine monophosphate-activated protein kinase (AMPK) signalling [30,31]. mTOR works with input from upstream pathways such as insulin, growth factors, and amino acids to sense cellular nutrient, oxygen, and energy levels [32]. AMPK inhibits the energy-intensive protein biosynthesis process, stimulates glucose uptake in skeletal muscle, and stimulates glycolysis [33]. mTOR and AMPK both negatively regulate autophagy through phosphorylation of Unc-51-like kinases ULK1 and ULK2 (mammalian homologues of Atg1) [34]. Autophagic conditions are activated through the dephosphorylation of ULK kinases as part of a complex including Atg13, Atg101 and FIP200, [35] which in turn will phosphorylate and activate Beclin-1 (mammalian homolog of Atg6), which is part of the autophagy inducible Beclin-1 complex containing PIK3R4(p150), Atg14L and the class III phosphatidylinositol 3-phosphate kinase (PI(3)K) Vps34 [36]. These two complexes (ULK and Beclin-1) then migrate to the initiation site of the autophagosome (the phagophore) [37]. The autophagosome is a double-membraned vesicle that is indicative of autophagy, which envelops the protein or organelle targeted for destruction. The formation of the autophagosome begins at the endoplasmic reticulum [38], with the construction of the omegasome (so called due to its shape resembling the Greek letter omega (Ω)). The omegasome is a subcomponent of the ER and consists of a lipid bilayer enriched in phosphatidylinositol 3-phosphate (PI(3)P) [39]. The phagophore elongates from the omegasome to engulf the target of autophagy and encircles it to become the autophagosome [40]. In mammals, the autophagosome size can vary from 500 nm to 1500 nm [41] and can be observed via several microscopic techniques, the most common of which is tracking of LC3 [42]. LC3 is a protein that is essential to autophagosome formation, LC3 (Atg 4) is cleaved by Atg 4, and this cleavage is required for the terminal fusion of an autophagosome with its target membrane, and LC3 remains associated until the last moment before its fusion. LC3 staining using immunohistochemistry reveals puncta staining under the fluorescent microscope when autophagy is activated as the LC3 is recruited to the autophagosomes. LC3 can also be monitored using western blotting analysis by tracking the cleavage of LC3BI to LC3BII [43]. Once the autophagosome has formed and contains the items to be degraded, it eventually fuses with a lysosome from what is known as the autolysosome. Inside the autolysosome, the contents, including the LC3 on the inner side, are degraded to their subsequent building blocks, in the case of proteins that would be amino acids [44].

The control of autophagy is essential in many disease states. The activation or inhibition of autophagy can be therapeutic depending on the disease, especially as autophagy may be cytoprotective or cytotoxic depending on the circumstances surrounding the induction of autophagy. For example, in the case of cancer, autophagy can protect cancer cells inside a tumour’s microenvironment that does not have access to nutrients and oxygen before angiogenesis [45]. At the same time, the activation of excessive autophagic flux can induce autophagic cell death.

## 2. Roles of Polyphenols in Autophagic Adaptation for Healthy Aging

### 2.1. Polyphenol-Mediated Autophagy in Cancer

The term cancer involves uncontrolled cell division due to complex factors, including mutations, epigenetic modifications, and environmental influences that change the behavioural pattern of the cell [46]. Although decades of study have given a broad understanding of fundamental mechanisms, with the advancement of science, new insights are often being identified through which cancer cells proliferate and evade the immune system. Moreover, during the therapeutic intervention, the cancer cells become chemoresistant towards drug treatment due to the invoking of intracellular mechanisms, including autophagy, to protect the host indicating the need for mechanism-based therapy.

In cancer, autophagy acts as a double-edged sword, both contributing to and suppressing the progression of the disease. Initially, autophagy was regarded to have tumour-suppressive functions, which could be identified from the genetic knockout, transfection studies of various autophagy-related genes in different cancer cells and experimental animals [47,48,49,50,51,52,53,54,55,56,57,58]. On the contrary, as the cancer progresses, attenuation of the autophagic process seems to be highly beneficial in the suppression of cancer as the autophagy pathway feeds the tumor cells upon hypoxia signalling and inhibits apoptosis [59,60,61,62,63]. In short, autophagy, whether beneficial or detrimental, depends on the type, stages of cancer cells, and stress involved [64]. Based on the available reports, drugs alone or in combination modulating autophagy are under clinical trials aimed to alleviate disease conditions.

Various polyphenols have shown anticancer efficacy against different types of tumors both in vitro and in vivo via autophagy modulation. Herein, we have selected several polyphenols and discussed their anticancer properties. Although the polyphenols exert anticancer efficacy through various mechanisms and numerous publications are available, only the studies related to autophagy are reviewed in the following section.

#### 2.1.1. Luteolin

Luteolin (3′,4′,5,7-tetrahydroxyflavone) is widely observed across the plant kingdom, and the plants rich in the compound are commonly used in traditional medicinal practices for hypertension, inflammation, and cancer [65]. Luteolin showed an increase in autophagy in human colon cancer cells with wild-type p53 but not in p53 mutant cells, indicating that luteolin induces autophagy in a p53-dependent manner with a significant increase in ER stress [66]. Luteolin-induced autophagy-mediated apoptotic cell death might be due to the rise in Nrf-2 expression by DNA demethylase and the subsequent interaction between Nrf-2 and p53 [67]. Additionally, luteolin epigenetically decreases the CpG methylation of the Nrf-2 promoter by inhibiting DNA methyltransferase (DNMT) and histone deacetylase (HDAC) expression resulting in Nrf-2 activation in colon cancer cells [68]. In contrast, luteolin-induced autophagy in p53-null Hep3B cells was cytoprotective. This may be due to the cell type (lack of p53) or concentration dependence, where higher concentration leads to lethal autophagy, and lower concentration exhibits cytoprotective autophagy [69]. In hepatocarcinoma cells (SMMC-7721, Huh7), luteolin treatment alone or co-treatment with tumour necrosis factor (TNF)-related apoptosis-inducing ligand (TRAIL) induced autophagic flux, promoted cell cycle arrest and sensitised the cells to apoptosis, via JNK-mediated death receptor 5 (DR5) expression [70,71]. 

Luteolin inhibited anchorage-independent colony growth and promoted cell cycle arrest at the G1 phase, apoptosis in lung cancer cells through regulation of peIF2α/CHOP and JNK pathway in a time-dependent manner. Further, luteolin also showed an increase in beclin-independent non-canonical autophagy mechanism accompanied by the suppression of LIM kinase-1 and related signalling pathway (p-LIMK1, p-cofilin and Ki-67) [72,73]. LIMK1, together with the downstream effector’s cofilin and Ki-67, is involved in the tumour cell filopodia formation resulting in migration, invasion, and metastasis [74]. Luteolin also exhibited selective apoptosis in cancer cells. Like normal keratinocytes (NHKs) are resistant to luteolin. Still, primary squamous cell carcinoma cells (MET1) showed higher sensitivity than metastatic (MET4) cells, which could be due to the contribution of the autophagic process as a survival mechanism. However, treatment with chloroquine, the autophagy inhibitor, along with luteolin, exhibited an increase in cytotoxicity in metastatic cells [75]. Combinatorial treatment of luteolin with silibinin inhibited angiogenic network formation, cell migration, invasion, and rapamycin-induced autophagic activity in glioblastoma (U87MG and T98G) cell suppression of PKCα. It increased the expression of the tumour suppressor miR-7-1-3p [76]. In ovarian cancer cells, luteolin treatment enhanced the sensitivity of the cells towards cisplatin treatment by suppressing poly (adenosine diphosphate (ADP)-ribose) polymerase-1 (PARP-1)-mediated autophagy [77]. PARP-1 has been reported to induce autophagy by activating liver kinase B-1 (LKB-1), AMPK and downregulating mTOR [78]. The available reports indicate that luteolin selectively targets the cancer cells and modulates autophagy to exert anticancer effects depending on the stress condition.

#### 2.1.2. Epigallocatechin Gallate

Epigallocatechin gallate (EGCG) is the most abundant green tea polyphenol with numerous health benefits [79,80,81,82]. In colorectal carcinoma cells (HT-29), EGCG induced autophagy to inhibit the proliferation of cells through the regulation of the glycerophospholipid metabolic pathway [83]. However, in HCT116 cells, EGCG treatment prevented TRAIL-induced apoptosis by stimulating autophagic flux and downregulation of death receptors (D4, D5). In comparison, inhibition of autophagy by chloroquine sensitised the cells towards TRAIL-induced apoptosis upon EGCG treatment [84]. EGCG synergised the therapeutic efficiency of irinotecan, a drug clinically used to treat colorectal cancer by inhibiting migration, invasion and inducing DNA damage, cell cycle arrest. In addition, the combinatorial administration of compounds was found to influence the cleavage of ataxia telangiectasia-mutated (ATM) and p-ATM leading to severe DNA damage in cells. Further, the synergistic action could increase apoptosis by the augmentation of autophagy [85]. The induction of autophagy could be due to the activation of AMPK by ATM, resulting in the suppression of mTORC1 [86]. Combinatorial treatment of EGCG with cisplatin or oxaliplatin synergistically inhibited the survival of colorectal carcinoma cells (DLD-1 and HT-29 cells) by inducing autophagy, whereas knockdown of ATG-5 reversed the effect [87]. Treatment of EGCG to CC-8 cells increased the sensitivity towards radiation and inhibited cell proliferation by inducing Nrf-2-mediated autophagy [88].

EGCG at a low concentration inhibited proliferation and regulated autophagy-related proteins by inhibiting PI3K/AKT/mTOR pathway to induce apoptosis in bladder cancer cells. Further, knockdown of ATG5 could reverse the effect of EGCG-induced apoptosis, confirming the role of EGCG in autophagy induction [89]. EGCG treatment could also sensitise the gefitinib-resistant non-small cell lung cancer towards drug by inhibiting Raf/MEK/ERK-mediated pro-survival autophagy, which occurred in cells upon gefitinib treatment [90]. In breast cancer cells (4T1), EGCG inhibited the expression of HIF-1α and glucose transporter 1 (GLUT1) and induced autophagy in a dose-dependent manner. The induction of autophagy was due to the inhibition of the HIF-1α-mediated glycolytic pathway by EGCG that helps turn on the autophagy process during starvation [91]. Studies also indicate that knockdown of HIF-1α reduces glycolytic metabolism and increases cell death [92]. In silico analysis suggests that EGCG could directly interact with LC3-I, inhibit dimerization, and promote LC3-II production, thus inhibiting HepG2 cell proliferation [93]. In cisplatin-resistant oral cancer cells (CAR cells), EGCG triggered apoptosis by inhibiting AKT/STAT3 signalling and activating autophagy. In addition, EGCG also downregulated the gene and protein expression of multidrug resistance-1 (MDR-1) to promote apoptosis in cells upon drug treatment [94]. The combined delivery of EGCG and two different proteasome inhibitors bortezomib and MG132 displayed different effects on prostate cancer cells (PC3). EGCG caused resistance in PC3 cells towards bortezomib treatment by preventing proteasome inhibition and induction of ER stress. However, EGCG and MG132 treatment effectively activated apoptosis and showed cytotoxicity in cells. Further, in the presence of bortezomib, EGCG triggered pro-survival autophagy to rescue the cells from drug treatment, which was inhibited in the presence of MG132 [95]. In osteosarcoma conditions, EGCG inhibited the pro-survival autophagy induced by doxorubicin to enhance the drug’s effect. The inhibition of autophagy was due to the targeting of lncRNA SOX2OT V7 and partial inhibition of Notch3/DLL3 signalling, which could further increase the sensitivity of the cells to chemotherapeutic drugs [96]. Similar results were also reported in Hep3B cells, where the genetic knockdown of ATG-5, beclin-1, further facilitated the antitumour activity of EGCG and doxorubicin [97]. However, the same combination loaded by polydopamine coating ZIF-8 showed chemo-photothermal activity by inducing autophagy and could ablate tumours in a mouse HeLa tumour model [98] indicating EGCG targets autophagy depending on the cellular need to improve the chemotherapeutic efficiency.

#### 2.1.3. Resveratrol

The phytoalexin resveratrol (3,4′,5-trihydroxy-trans-stilbene) occurs naturally in a wide range of plants, including grapes and berries with profound anticancer activities [99]. Furthermore, many plants cultivated in Asia as folk medicines, such as *Polygonum cuspidatum*, *Rhodomyrtus tomentosa*, *Rheum undulatum*, and *Melaleuca Leucadendron,* also contain resveratrol contributing to their medicinal properties [100,101,102,103]. Resveratrol treatment mediated both canonical (Beclin-1 dependent) and non-canonical autophagy (independent of Beclin-1, ATG-5), increasing LC3B lipidation and reducing apoptosis in A549 cells by activating Sirt-1, p38-MAPK pathway and inhibiting Akt/mTOR. Use of 3-methyladenine or Sirt-1 inhibitor (nicotinamide) enhanced the antitumour activity of resveratrol [104,105]. Further, resveratrol sensitised A549 cells towards TRAIL-mediated cell death by suppressing Akt/NF-κB, increasing cytochrome-c translocation and mitochondrial dysfunction independent of autophagy, p53 mechanism [106]. Resveratrol inhibited breast cancer stem cells (BCSC; isolated from MCF-7 cells) and caused cell cycle arrest by inducing autophagy via the suppression of the Wnt/β-catenin signalling pathway [26]. During cancer, Wnt/β-catenin signalling is deregulated, resulting in cell proliferation and shows cross-talk with autophagy through negative regulation. On the other hand, autophagy activation inhibits the pathway by degrading Dvl or β-catenin [107,108,109]. Opposed to BCSC cells, resveratrol could potentiate long-term apoptosis and senescence induced by acute treatment with doxorubicin but with cytoprotective autophagy activation to resist apoptosis, which was reversed upon inhibition of autophagy in MCF-7 cells [110]. Resveratrol treatment in cancer cells downregulated TP53-induced glycolysis and apoptosis regulator (TIGAR) expression, resulting in the subsequent reduction of glutathione, ROS accumulation, and activation of protective autophagy, wherein use of appropriate autophagy inhibitors induced cell death, suggesting combination therapy for cancer treatment [111]. Although TIGAR inhibits the glycolytic pathway, increased protein expression has been reported in cancer cells, indicating that it activates survival mechanisms through other mechanisms. Reports suggest that knockdown of TIGAR enhances drug-induced ROS generation and directly inhibits mTOR activation resulting in autophagy and subsequent increase in apoptosis [112]. Resveratrol treatment also induced the level of autophagy-related proteins, including LAMP1, Atg7, LC3B, PINK1 and PARK2, accompanied by an increase in the cellular lysosome, ROS, and impairment of energy metabolism [113]. In human ovarian cells, resveratrol treatment triggers autophagy with subsequent reduction in apoptosis, which was further reversed by inhibition of autophagy [114]. Transcriptomic and microRNA profiling studies have shown that resveratrol treatment modulates several genes and microRNAs involved in the locomotion, migration, and invasion in ovarian cancer cells. Further, resveratrol triggers autophagy via upregulating the expression of the tumour suppressor ARH-1 and inhibition of STAT3 activation [115,116]. Resveratrol treatment blocks glucose uptake in ovarian cancer cells, triggers starvation-like signalling response (inhibition of Akt/mTOR) and upregulates LMP level resulting in the cytosolic translocation and activation of cathepsin L to promote autophagy-mediated cell death [117,118]. Resveratrol was found to inhibit the stem cell-like characteristics, migration, and invasion of pancreatic cancer both in vitro and in vivo by Nrf-2-mediated mitigation of nutrient-deprivation autophagy factor-1 (NAF-1) [119,120]. NAF-1 has been previously reported to inhibit beclin-1-dependent autophagy and promote longevity indicating that resveratrol induces autophagy during pancreatic cancer to exhibit anticancer effect [121]. Resveratrol could sensitise TRAIL-resistant renal cancer cells (786-0 and OS-RC-2) towards TRAIL treatment and promote apoptosis via upregulation of autophagy [122]. In human cholangiocarcinoma cell lines (KKU-213 and KKU-100), conditioned medium from cancer-associated fibroblasts (CAFs) stimulated the secretion of IL-6, which further helped in the migration of cancer cells. Further, the E/N cadherin switch was activated that acts as a hallmark for the invasive behaviour of cholangiocarcinoma cells accompanied with inhibition of autophagy. However, resveratrol treatment inhibited cell migration by suppressing IL-6 secretion, inducing N/E cadherin switch (mesenchymal to epithelial transition) and augmenting autophagy [123]. In the bone cancer pain induced model of Sprague Dawley rats, resveratrol administration significantly downregulated the expression of acid-sensing ion channels 3 (ASIC3) to alleviate the pain. Further, in SHSY-5Y cells transfected with ASIC-3, resveratrol treatment in a dose-dependent manner regulated ASIC-3 expression and activated the AMPK-SIRT1-autophagy signal pathway [124]. Resveratrol induced autophagy via AMPK/Akt/mTOR pathway and exhibited cytotoxicity in a caspase-dependent manner in cisplatin-resistant oral cancer cells and pancreatic cancer cells [125,126]. Resveratrol was reported to induce protective autophagy in HGC-27 cells, which was attributed to increased intracellular dihydroceramide (ceramide metabolic precursor) levels due to the inhibition of dihydroceramide desaturase enzymatic activity [127]. Similar results were also observed in melanoma B16 cells, where resveratrol treatment induced protective autophagy mediated via Ceramide/Akt/mTOR pathway [128]. Resveratrol induced both intrinsic (increase in Bax/Bcl-2) and extrinsic (caspase activation) apoptotic pathways in the human promyelocytic leukemia cell line (HL-60). Further, resveratrol activated the LKB1-AMP pathway, inhibited PI3K/AKT signalling and contributed to mTOR inhibition for autophagy induction, which further triggered cell death [129]. In human colorectal cancer cells, resveratrol treatment triggered autophagy and induced apoptosis dependent on class III PI3K (Vps34). Further, genetic knockout of Lamp2b abrogated autophagolysosome formation and cell death induced by resveratrol [130].

#### 2.1.4. Curcumin

Curcumin [1,7-Bis(4-hydroxy-3-methoxyphenyl)-1,6-heptadiene 3,5-dione], obtained from *Curcuma longa* is one of the most studied phytochemicals for a range of biological functions including anti-inflammation, neuroprotective, antidiabetic, and anticancer ones [131]. In human colon carcinoma cells (HCT-116 cells), curcumin-mediated cytotoxicity was attenuated by the expression of c-Jun NH2-terminal kinase (JNK)/stress-activated protein kinase associated protein 1 (JSAP1), a scaffold protein involved in JNK signaling pathway and lysosomal transport with an increase in autophagy. Knockout of JSAP1 attenuated autophagic flux and sensitised the cells towards curcumin treatment [132]. Curcumin treatment in colorectal cancer cells evoked pro-survival autophagy via cathepsin C, the lysosomal acid hydrolase, to offer chemotherapeutic resistance in cancer cells. However, silencing of cathepsin C followed by curcumin treatment ER stress, autophagic dysregulation, and cathepsin B mediated lysosomal permeabilization resulting in apoptosis [133]. Further, cytoprotective autophagy is also increased through the transcriptional activation of transcription factor EB (TFEB) in HCT-116 cells by curcumin. An increase in TFEB activity was due to mTOR inhibition, which negatively regulates the transcription factor [134]. On the contrary, curcumin-induced autophagy promoted tumour growth inhibition via downregulation of yes-associated protein (YAP), which is involved in cell proliferation, promoting malignant cell transformation, and controlling autophagy has been reported in HCT116 cells [135,136,137]. Further, curcumin mitigated the mTORC1 kinase activity in Caco-2 cells and significantly attenuated the expression of insulin receptor substrate-1 (IRS-1), p-AKT, p-PRAS40, and Rapt, or resulting in the simultaneous activation of the MAPK pathway. Curcumin treatment upregulated the expression of LKB1, AMPK, and AMPK-mediated phosphorylation of TSC2, indicating the role of autophagic tumour suppression [138]. Curcumin was also found to induce autophagy in LGR5(+) colorectal cancer stem cells and promote apoptosis. In addition, RNASeq analysis showed that curcumin treatment inhibited the oncogenic TFAP2A-mediated ECM pathway by negatively regulating the associated genes, including *COMP, LAMA5,* and ITGA1, wherein TFAP2A expression is associated with poor prognosis in cancer patients [139]. Previous reports have indicated that TFAP2 inhibition promotes ferroptosis and inhibits the migration and invasion of cancer cells and there exists a cross talk between autophagy and ferroptosis [140,141]. In line with this, curcumin has also been reported to induce ferroptosis in non-small cell lung carcinoma mediated through autophagy [142]. Combinatorial treatment with curcumin and gefitinib inhibited epidermal growth factor receptor (EGFR) by attenuating Sp1, and HDAC1 binding induced EGFR transcription activity resulting in autophagy and sensitised A549 cells from gefitinib resistance [143]. Moreover, curcumin mediates human GD3 synthase (hST8Sia I) gene transcription through AMPK activation and induces autophagy. Enhanced hST8Sia I gene expression is further associated with an increase in ganglioside GD3 expression, which plays an important role in autolysosome formation and maturation by interacting with LC3 and LAMP1 [144,145]. Curcumin has been shown to induce cell cycle arrest, autophagy, autophagy flux, cellular senescence and enhanced apoptosis in cervical cancer cells [146], while promoting protective autophagy and apoptosis through the suppression of AKT/mTOR/p70S6K signalling pathway in ovarian cancer cells. Combinatorial treatment with the autophagy inhibitor chloroquine suppressed the autophagy and further enhanced curcumin-mediated cell death [147]. Curcumin attenuated HepG2 cell proliferation and induced apoptosis by autophagy-mediated suppression of glypican-3 (GPC-3) and wnt/β-catenin signalling pathway. GPC-3 is involved in proliferation, invasion, metastasis, and is highly expressed in hepatocellular carcinoma tissues [148,149]. Further, knockdown of GPC-3 has been reported to inhibit the proliferation of Huh-7 cells by the downregulation of YAP, which could have been the reason for the induction of autophagy by curcumin [150]. Combinatorial treatment of curcumin and docetaxel in oesophagal squamous cell carcinoma mitigated the migration, invasion of cancer cells and further induced apoptosis, autophagy-mediated through PI3K/AKT/mTOR pathway, where inhibition of autophagy was found to increase the apoptosis indicating the activation of pro-survival autophagy upon drug treatment [151]. Curcumin was reported to epigenetically restore the expression of miR-143/miR-145 cluster by reducing CpG methylation of miR-143 promoter and reducing the expression of DNMT1, DNMT3B in prostate cancer cells. miR-143 is regarded as a tumour suppressor and inhibitor of cancer cell metastasis, with an additional role in regulating autophagy. In line with the report, upregulation of miR-143 by curcumin inhibited autophagy in prostate cancer cells via downregulating ATG2B and sensitised the cells towards irradiation [152,153].

#### 2.1.5. Quercetin

Quercetin (3,3′,4′,5,7-pentahydroxyflavone), widely found in tea, red wine, broccoli, apple, and in many other products, has several immunomodulatory effects in humans. Quercetin inhibited A549 cell proliferation and enhanced TRAIL-mediated cell death by activating the autophagy process via the SIRT-1/AMPK pathway in a dose-dependent manner [154,155]. Similar to curcumin, quercetin also promoted lysosomal activation, autophagy and ferroptosis through the activation of TFEB, induced p53-independent cell death [156], and inhibited cancer cell migration and invasion by inhibiting JAK2/STAT3 pathway in hepatocellular carcinoma cells [157]. Quercetin treatment in pancreatic cancer cells in combination with gemcitabine downregulated Hsp70 and promoted autophagy and apoptosis [158]. Overexpression of Hsp70 is associated with inhibition of autophagy and progression of carcinogenesis [159,160]. The receptor for advanced glycation end products (RAGE) is being overexpressed in various cancer cells, including pancreatic cancer, resulting in the development of drug resistance. Quercetin co-treatment with gemcitabine in MIA Paca-2 and MIA Paca-2^GEMR^ (gemcitabine resistant) cells induced cytotoxicity by increasing chemosensitivity, suppressing RAGE expression and inducing autophagy via RAGE/PI3K/AKT/mTOR axis [161].

Further, quercetin inhibited breast cancer metastasis both in vitro and in vivo by suppressing glycolysis and inducing autophagy via Akt/mTOR pathway [162]. In ovarian cancer cells, quercetin treatment through ER-stress mediated p-STAT3/Bcl-2 axis induced cytoprotective autophagy, which was attenuated with autophagy inhibitors [163]. Co-treatment with quercetin and chloroquine induced oxidative ER stress followed by calcium imbalance and promoted cell death in glioma cells. In addition, the co-treatment further enhanced quercetin-mediated LC3B expression along with an increase in p62, indicating that lysosomal inhibition plays a significant role in the death of glioma cells [164]. In osteosarcoma cells, quercetin treatment augmented NUPR-1-mediated autophagic cell death accompanied by the increase in ROS level [165]. Evidence indicates that NUPR-1 helps in the transcriptional activation of genes involved in autophagosome formation and is also involved in cell cycle regulation, DNA repair and apoptosis [166,167]. Quercetin induced intrinsic mitochondrial apoptosis and exerted DNA damage by activating the ATM-CHK-p53 pathway in Jurkat T cells and induction of cytoprotective autophagy, whereas inhibition of autophagy potentiated BAK-dependent apoptotic death [168]. Quercetin treatment promoted the expression of Apoptosis-Stimulating Protein of p53-1 (ASPP-1), an activator of p53 through the activation of the zinc-finger nuclear transcription factor Early Growth Response-1 (EGR-1) and exhibited cytotoxicity in human colorectal carcinoma cells. Further, knockdown studies indicated that EGR-1/ASPP-1 downregulated cytoprotective autophagy activation upon quercetin treatment and enhanced apoptosis in the cancer cells [169]. Autophagy inhibition could be due to the ability of ASPP-1 to inhibit large tumour suppressor-1 (LATS-1) and prevent the phosphorylation and proteosome degradation of YAP and TAZ (transcriptional coactivator with PDZ-binding motif), as YAP negatively regulates the autophagy process [170].

Quercetin induced complete autophagy in Burkitt lymphoma by inhibiting PI3K/AKT/mTOR resulting in the reduction of c-Myc, which plays a critical role in the disease [171]. Du et al. (2015) have demonstrated that the combination of quercetin and transfecting human gastric cancer cell lines with miR-143, which was initially downregulated in the cancer cells, inhibited autophagy via GABARAPL1 (ATG-8) suppression and increased the chemosensitivity of the cells towards quercetin [172].

#### 2.1.6. Silibinin

The hepatoprotective drug silibinin is isolated from *Silybum marianum* with reported anticancer effects [173]. In glioblastoma cells, silibinin induced autophagy through Akt/mTOR pathway, Hippo pathway (YAP) and mediated apoptosis through caspase-3, PARP cleavage. However, chloroquine treatment inhibited autophagy and further enhanced the level of apoptosis, indicating activation of cytoprotective autophagy by silibinin [174]. Additionally, the nuclear translocation of apoptosis-inducing factor (AIF) accompanied by activation of autophagy by the suppression of glycolysis causing ATP deficiency in glioma cells has been reported. Silibinin-induced autophagy increased the mitochondrial accumulation of Bcl-2/adenovirus E1B 19-kDa-interacting protein 3 (BNIP3) by augmenting H_2_O_2_ levels in cells via depletion of glutathione. In addition, silibinin also induced autophagy-dependent phosphorylation of p53 resulting in apoptosis of glioma cells [175]. Involvement of autophagy-mediated BNIP3 activation was also observed in breast cancer cells [176]. Silibinin has been reported to induce AIF nuclear translocation in a concentration-dependent manner, which is also dependent on the expression of estrogen receptor α/β (ER- α/β). Silibinin treatment significantly attenuated the mitochondrial and extra-mitochondrial expression of ER- α, while a reversal in effect was observed in the case of ER-β. ER-α downregulation is further accompanied by autophagy activation, increasing AIF translocation and apoptosis [177]. Si et al. (2020) reported that silibinin inhibits EMT transition and metastasis by augmenting the expressions of E-cadherin and suppressing matrix metalloproteinases respectively in breast cancer cells. In addition, silibinin disturbed mitochondrial biogenesis by downregulating mitochondrial transcription factor A (TFAM), PGC-1γ and Nrf-2 accompanied with inhibition of the NLRP3 inflammasome activation and metastasis [178]. Silibinin-induced mitochondrial fission showed impaired mitochondrial dynamics, mitochondrial biogenesis in breast cancer cells accompanied by autophagy and mitophagy. Suppression of mitophagy through knockdown of Pink and Parkin-1 further enhanced apoptosis in varying degrees in MCF-7 (ER-positive) and MDA-MB-231 (ER-negative) cells indicating silibinin-induced mitophagy is cytoprotective. However, studies with agonists and antagonists show that the ERs pathway is not involved in activating mitophagy by silibinin but acts in parallel to exhibit the effect [179]. ER-α activation augments protective ROS/RNS generation in cells and inhibits autophagy resulting in cell survival. At the same time, silibinin treatment was reported to inhibit ER-α and promote ER-β resulting in autophagy and apoptosis. However, ER-β does not influence silibinin-induced autophagy, indicating that both pathways act parallel [180,181]. In HeLa/SW1990 cells, silibinin induced JNK-mediated p53 activation resulting in autophagy and apoptosis [182,183]. Silibinin attenuated the cancer cell metastasis and epithelial to mesenchymal transition through the autophagy-dependent (AMPK/mTOR) downregulation of the Wnt/β-catenin pathway. β-catenin has been previously reported to be degraded through the autophagy-lysosome system, which might have resulted in suppressing the signalling pathway [184,185]. In human colon cancer cells (both primary and metastatic), silibinin treatment activated both extrinsic (TRAIL receptors, caspase-8, 10, Bid) and intrinsic (cytochrome-c, caspase-9) apoptotic pathways associated with induction of cytoprotective autophagy [186]. Human fibrosarcoma cells treated with silibinin showed an increase in ROS-mediated expression of p38 and NFκB, resulting in autophagy and cell death [187]. Silibinin protects A431 cells from UVB induced apoptosis through activation of autophagy. UVB irradiation has been reported to downregulate the IGF-1R-PI3K-Akt axis, which conversely regulates autophagy. Silibinin downregulates the expression of IGF-1R, with subsequent autophagy activation and protect against skin cancer [188]. Similarly, upon silibinin treatment, mice exposed to UVB promoted epidermal autophagy and decreased p53 activation and protected against skin erythema [189].

As discussed earlier, the polyphenols can either inhibit or induce autophagy depending on the conditions, which may also affect the sensitivity of the cells to apoptosis. Hence, a combinatorial approach with the use of autophagy inhibitors is needed to overcome the barrier. Further, most of the polyphenols exert a synergistic effect and mitigate the chemoresistance when supplemented along with already existing anticancer drugs. This approach can also be implemented, which would reduce the need for high-dose administration when administered alone and mitigate the adverse effect of synthetic drugs [190].

### 2.2. Polyphenol-Mediated Autophagy in Diabetes Mellitus (DM) and Associated Complications

Diabetes and its associated complications are regarded as chronic metabolic conditions and have become a considerable threat worldwide due to increased longevity and poor lifestyle. Diabetes mellitus is accompanied by several other complications, including diabetic nephropathy, diabetic retinopathy, diabetic neuropathy, and diabetic cardiomyopathy [191]. Under hyperglycemic conditions, several metabolic pathways are modulated, resulting in the generation of ROS, advanced glycation end products (AGEs), leading to oxidative stress, ER stress, inflammation, and impaired autophagy [192,193,194,195]. Accumulating evidence shows a reduction in autophagy during diabetes conditions, and reports have pointed out that increased insulin resistance downregulates the genes involved in the autophagic pathway, abrogating the degradation of lipid droplets, a process termed lipophagy, which in turn contributes to insulin resistance [196,197,198,199,200]. In contrast, pancreatic samples from diabetic individuals showed increased dead β cells with massive vacuole overload, indicating autophagy-mediated apoptosis [201], implying autophagy, like in any other disease, can play both beneficial and detrimental effects in diabetes.

Polyphenols with high antioxidant and anti-inflammatory potential, besides influencing glucose uptake, metabolism, inhibition of glucose absorption and improving insulin secretion, also modulate autophagy under hyperglycemic conditions [202,203]. In individuals with T2DM, insulin and human islet amyloid polypeptide (hIAPP) co-express in the pancreatic beta cells, forming amyloid deposits to disrupt the cell function. Resveratrol treatment in INS1 cells (isolated from rat insulinoma) overexpressing hIAPP reduced amyloid formation through activation of autophagy and restored insulin secretion [204]. Resveratrol administration in streptozotocin (STZ)-induced diabetic mice improved muscle function, inhibited ubiquitin–proteasome system, and autophagy in muscles. Histopathological observation showed improved mitochondrial content, preventing mitochondrial fission, fusion (downregulation of p-Drp-1, Fis-1), and increased mitochondrial biogenesis (Nrf-1, Pgc-1α). In addition, resveratrol treatment attenuated muscle mitophagy by downregulating BNIP3L, PINK1, and Parkin expression [205]. In STZ-induced diabetic mice, resveratrol extenuated glucose and insulin tolerance and mitigated oxidative stress parameters. Further, in beta cells of pancreatic islets of the type-1 diabetic mice (T1DM), resveratrol upregulated CXC chemokine ligand 16 (CXCL16) expression, which works as a scavenger receptor for ox-LDL accompanied with reduction of ox-LDL expression and inhibition of coagulation cascade by downregulating tissue factor (TF). In addition, resveratrol mitigated autophagy in the beta cell of pancreatic islets in T1DM and restored the islets architecture [206].

Resveratrol enhanced the interaction between AMPK and JNK1 and JNK-1-mediated interruption of Beclin1-Bcl-2 complex in cardiac myocytes cells exposed to high glucose content to induce autophagy and inhibit apoptosis. Further, it also downregulated the expression of connexin-43 (a negative regulator of autophagy) and apoptosis-associated marker proteins (Bax) [207,208,209]. Additionally, resveratrol enhanced the DNA-binding ability of FOXO1 to the promoter of Rab7 through the SIRT1-dependent pathway and activated autophagy in the myocardium of diabetic mice [210]. Rab7 is involved in the regulation of autophagy, and its knockdown results in an impaired autophagy process [211]. In addition, under ischemic/reperfusion conditions, resveratrol protected the myocardium by inducing autophagy and blocking inflammatory cytokines [212].

In a mouse model (C57BL/KsJ db/db) of diabetic nephropathy and human podocytes treated with high glucose, resveratrol induced autophagy and inhibited apoptosis. Silencing of Atg-5 or the use of autophagy inhibitors reversed the protective effects indicating a resveratrol-mediated protective effect through the autophagy pathway [213]. In T2DM nephropathy rats, resveratrol treatment downregulated the expression of pro-inflammatory cytokines and ameliorated renal dysfunction, with an increase in Sirt-1, hypoxia-induced (BNIP3) autophagy activity [214]. Under chronic diabetic nephropathy conditions in mice, resveratrol administration protected the diabetic kidney by inducing autophagy and attenuated glomerular injury. Induction of autophagy and suppression of apoptosis occurred with the significant upregulation of microRNA-18a-5p. Knockdown of Atactic Telangiectasis Mutation (ATM), a target gene of microRNA-18a-5p, increased autophagy and inhibited apoptosis [215]. On the contrary, resveratrol alleviated diabetic nephropathy conditions by attenuating oxidative stress and insulin resistance and augmenting AMPKα/mTOR-mediated autophagy induction in STZ-induced diabetic rats. In addition, resveratrol also improved lipid metabolism through the upregulation of lipolysis related proteins (PPARα, CPT-1) and downregulating lipogenic related proteins (SREBP-1c, ACS) [216]. The silencing of sterol regulatory element-binding proteins (SREBPs) has been reported to attenuate PERK-mediated apoptosis [217]. In T1DM, resveratrol treatment significantly attenuated testicular apoptotic cell death by preventing oxidative damage (inhibition of 3-nitrotyrosine, 4-hydroxynonenal) and augmentation of the Nrf-2-mediated antioxidant pathway. In addition, resveratrol downregulated the expression of p-GSK-3β, p-Akt accompanied with the upregulation of the negative regulators of Akt (PTEN, TRB3 and PTP1B. Further, resveratrol also promoted autophagic degradation of Kaep-1, thereby disrupting Kaep-1-Nrf-2 interaction and activating Nrf-2 [218].

Diabetic retinopathy conditions in Müller cells induced by high glucose were attenuated by the treatment with EGCG resulting in enhanced autophagy and reduced apoptosis [219]. Oxidative stress-mediated mitochondrial dysfunction in the heart of T2DM Goto–Kakizaki rats and STZ-induced diabetic mice were significantly attenuated by the administration of EGCG accompanied with FOXO1/SIRT-1-mediated autophagy [220,221]. Lipid accumulation in pancreatic beta cells results in the dysfunction of cells causing impaired insulin secretion and development of diabetes [222]. Kaempferol treatment to RIN-5F cells upon exposure to palmitic acid inhibited intracellular lipid accumulation with reduced expression of proteins localised on the surface of lipid droplets (Plin2 and Plin3). Kaempferol abolished palmitic acid-induced lipid droplets by enhanced lipophagy through the AMPK/mTOR pathway. Induced autophagy by kaempferol also attenuated ER stress and restored β-cells function [223,224].

Silibinin treatment protected INS1 cells from the toxic effects of TNF-α and IL-1β by augmenting autophagy through the activation of ER-α/β [225]. Under high-glucose conditions, silibinin maintained the viability of HUVEC cells by abolishing oxidative stress and increasing autophagy [226]. In STZ-induced diabetic mice, silibinin administration reduced apoptosis in pancreatic beta cells by modulating autophagy via SIRT-1 regulation [227]. Isohamnetin administration in T2DM rats restored adipose tissue and myofibrils architecture and downregulated mTOR, IGF1R and AKT in the skeletal muscle and adipose tissue. In addition, an increase in miR-1 and miR-1363 expression was also reported, associated with reduced expression of LncRNA-RP11-773H22.4 [228]. Moreover, diabetic nephropathy conditions were also significantly attenuated, accompanied with the epigenetic modulation (miR-15b, miR-34a and miR-633)-mediated activation of autophagy-related genes (FYCO1, ULK, TECPR1 and WIPI2) and autophagosome formation [229].

Baicalin administration was shown to reverse hyperglycemia-inhibited development of early chick embryos, and inhibit malformation of embryonic cardiovascular system and apoptosis through the suppression of oxidative stress and autophagy [230]. Punicalagin treatment improved the liver functions of type-2 diabetic mice (T2DM) and protected high-glucose-treated HepG2 cells through the Akt/FoxO3a signalling pathway [231]. In the T1DM rat model, the isoflavonoid puerarin downregulated FOXO-3a with subsequent reduction of the muscle atrophic markers Atrogin-1, Murf-1 and promoted muscle transformation from oxidative type (slow or type I fibres) to glycolytic type (fast or type II fibres). The suppression of FOXO-3a mediated Atrogin-1 downregulation. Furthermore, the compound treatment mitigated autophagy and enhanced Akt/mTOR signalling in L6 myotubes upon treatment with high glucose [232]. The A-type procyanidin oligomer cinnamtannin D1 protected pancreatic beta cells from glucolipotoxicity by activating and restoring impaired autophagy via the AMPK/mTOR/ULK1 pathway. Autophagy activation further induced Keap1/Nrf2 antioxidant pathway and attenuated ER stress (PERK, CHOP) and inflammation-related proteins (JNK, NFκB) and protected the cells from apoptosis. Similar results were also observed in the pancreas of diabetic mice (C57BKS db/db), indicating the protective effect of the compound [233].

In diabetic nephropathy conditions, dihydromyricetin promoted autophagy through the regulation of miR-155-5p and its target gene PTEN and PI3K/AKT/mTOR pathway under both in vitro and in vivo conditions [234]. Similarly, in high-glucose-induced cardiomyocytes and diabetic mice, dihydromyricetin inhibited miR-34a, resulting in the activation of autophagy, where complete inhibition resulted in impaired autophagy, indicating that a low level of miR-34a is needed to exhibit the protective effect of the compound [235]. In T2DM rats with erectile dysfunction, either icariside II treatment alone or with metformin improved erectile function, alleviated oxidative stress and attenuated mitochondrial autophagy through activation of PI3K-AKT-mTOR signalling pathway [236,237]. The citrus flavonoid naringin protected HUVEC cells from high-glucose/high-fat toxicity by attenuating the autophagic flux through the PI3K-Akt-mTOR pathway [238]. Delphinidin-induced autophagy mediated through AMPK protected pancreatic beta cells from high glucose injury [239]. Pelargonidin-3-O-glucoside derived from wild raspberry showed resistance against high glucose and high fat in HepG2 cells and enhanced glucose uptake through autophagy induction mediated by the activation of TFEB. In addition, the compound also showed an antidiabetic effect in db/db diabetic mice via the exact mechanism as observed in HepG2 cells. Pelargonidin-3-O-glucoside administration modulated the gut microbiota, especially with the increase in abundance of *Prevotella*, which is associated with increased glucose metabolism [240].

### 2.3. Polyphenols-Mediated Autophagy in Neurodegenerative Diseases 

Neurodegenerative diseases are a range of diseases of the brain, including Parkinson’s disease (PD) and Alzheimer’s disease, which result in neuron damage, brain atrophy, and consequently, the loss of cognitive or physical abilities. Several factors, including oxidative, endoplasmic stress, impairment in protein folding machinery, defects of damaged protein/organelle clearance mechanism, play key roles in the disease pathogenesis [241,242,243,244,245,246,247,248] and targeting these mechanisms alleviates the condition and offers neuroprotection. 

The wide range of polyphenolic compounds that are found in many kinds of berries, fruits, and vegetables with potent antioxidant capacity can activate and inhibit a vast number of pathways involved in these diseases [249]. A selection of polyphenolic compounds is discussed below with regards to modulating autophagy under neurodegenerative conditions.

#### 2.3.1. Phenolic Acids

Phenolic acids are one of the most common types of polyphenols. They are abundant in berries, almonds, coffee and tea, and whole grains [250,251]. Several phenolic acids, including ferulic acid, caffeic acid, caffeic acid phenyl ester, rosmarinic acid, p-coumaric acid, sinapic acid, cinnamic aldehyde, salicylic acid, acetylsalicylic acid, protocatechuic acid, gallic acid, tannic acid, homovanilic acid, syringic acid, and ellagic acid, have been reported as having neuroprotective potential [252].

Ferulic acid exhibited its antidepressant property by various mechanisms such as by enhanced level of monoamine oxidase (MAO) in the hippocampus and frontal cortex, serotonin and noradrenaline level on the hypothalamus [253,254]. Ferulic acid also enhanced antioxidant biochemicals like SOD, CAT, GPx in the cerebral cortex region while decreasing the level of TBARS in blood, hippocampus, and cerebral cortex in rodents [255]. Some studies have suggested that ferulic acid is responsible for the significant upregulation of BDNF, postsynaptic density protein (PSD95), and synapsin I level in mice’s prefrontal cortex and hippocampus [256]. Ferulic acid exhibited ameliorative potential in PD to attenuate the level of total protein, lipid peroxidase, IL-1β, IL-6, Bax/Bcl2 ratio, calcium-binding adaptor molecule (Iba-1) and GFAP hyperactivity [257,258].

Caffeic acid also plays a significant protective role in PD by decreasing the level of α-synuclein level [259] while the caffeic acid phenylethyl ester showed therapeutic potential against Huntington’s disease by reduction of striatal damage, immunoreactivity to glial GFAP, and lymphocyte common antigen (CD45) (markers of astrocyte and microglia activation) [260]. Furthermore, caffeic acid has been shown to activate JNK/Bcl-2-mediated autophagy which in turn reduces α-synuclein expression [261], and multiple other studies have demonstrated caffeic acid to be involved in the regulation of autophagic processes and preventing ER stress which is a critical player in neurological disease [262,263].

Chlorogenic acid exhibited therapeutic potential against depression by enhancing the enhancing synapsin-I expression (via 5-hydroxytryptamine receptors) and stimulation of axon and dendrite growth, promotion of serotonin release [264]. Chlorogenic acid also exhibited antiepileptic potential by reducing the lipid peroxidation and nitrite content and the mRNA expressions of *N*-methyl-d-aspartate receptor, metabotropic glutamate receptor 1 and metabotropic glutamate receptor 5 [265].

Multiple studies link chlorogenic acid to autophagy regulation regarding neurodegenerative diseases. Chlorogenic acid ameliorates the detrimental effects of amyloid-beta (Aβ_(25–35)_) in both SH-SY5Y cells and rats. Aβ_(25–35)_ treatment to SH-SY5Y cells was shown to induce increased autophagic flux and autophagosome production, putting pressure on cell metabolism and leading to neuron cell death [266]. Chlorogenic acid treatment of SH-SY5Y cells exposed to Aβ_(25–35)_ reduced LC3-I conversion to LC3-II, Atg-4 and Beclin-1 expression, suggesting that chlorogenic acid suppress the production of autophagosomes [267]. Furthermore, mTOR phosphorylation is affected by chlorogenic acid in SH-SY5Y cells exposed to Aβ_(25–35),_ indicating an increase in lysosomal activity. This suppression of autophagy and activation of lysosomal activity was also confirmed in mice [267].

Rosmarinic acid exhibited attenuation of seizures, mitigation of the oxidative stress, augmentation of the activity of defensive systems, reduction of MDA and nitrite content and increase of CAT activity; prevention of the hippocampal neuronal loss in CA1 and CA3 regions as well as reduction of the levels of free radicals and DNA damage in the kindling CF-1 [268,269]. Rosmarinic acid is also responsible for the downregulation of mitogen-activated protein kinase phosphatase-1, upregulation of BDNF. Rosmarinic acid also counteracted the stress-induced tauopathy by efficient suppression of the elevation of P-tau and insoluble P-tau formation. It also exhibited antiparkinson effects by preventing the loss of dopaminergic neurons in substantia nigra pars compacta, lowered iron reactivity, and attenuated MDA and nitrite levels in the midbrain [270,271].

Cinnamic aldehyde reversed various abnormalities such as exploratory behaviour, central ambulation and total ambulation-anxiety behaviour, rearing, grooming, immobility period by reducing COX-2 protein activity PGE2 concentrations in the frontal cortex and hippocampus [272].

Protocatechuic acid with its antioxidant potential attenuated the loss of neurons by inducing Nrf2-related factor 2 protein expression, upregulation of the expression of antioxidant enzymes such as heme oxygenase-1, SOD, CAT; decreasing MDA, NF-κB, and iNOS levels. Protocatechuic acid also exhibited its antiparkinson effect by increasing tyrosine hydroxylase and dopamine receptor D2 and reducing iNOS expression in striatum and midbrain [273]. 

#### 2.3.2. Flavonoids

Flavonoids have a long history of being used in medicine to treat various diseases [274]. They are a dominant class of medicinal agents due to their diversity, wide distribution, and ease of isolation. Flavonoids are essential building blocks for synthesising many medications and can also be used as natural products. Therefore, they play a crucial role in drug development and discovery [275]. More than 7000 flavonoids have been identified thus far in natural sources such as medicinal plants, vegetables, fruits, and wines. Flavonoids can attach to various bodily proteins transporters, enzymes, hormones, DNA, and also act as metal chelators and free radical scavengers due to their antioxidant potential [276]. Earlier literature has reported a plethora of pharmacological studies that suggest their utility in managing diabetes mellitus (DM), cancer, cardiovascular diseases, neurological disorders, inflammation, and microbial disease [276,277]. Recent research has found that consuming flavonoid-rich meals daily can help humans improve their cognitive capacities [278,279].

Furthermore, some flavonoids have been shown to slow the progression of Alzheimer’s disease (AD) pathology, owing to their capacity to alleviate cognitive deficits in various standard and transgenic preclinical animal models [279]. The interactions of flavonoids and their metabolites with different cellular and molecular targets are responsible for the positive benefits of foods high in flavonoids, such as chocolate, green tea, and blueberries [280]. Flavonoids’ interactions with receptors in the ERK and PI3-kinase/Akt signalling pathways, for example, have been shown to increase the expression of neuromodulatory and neuroprotective proteins, as well as the quantity and strength of various types of neurons [281,282]. Moreover, their favourable effects on the cerebrovascular system can improve individuals’ cognitive performance by increasing blood flow and stimulating neurogenesis in the brain. Several new mechanisms for flavonoids’ positive effects have lately been discovered [283]. Flavonoids slow the onset and progression of Alzheimer’s disease-like symptoms and other neurodegenerative illnesses, which could be due to the inhibition of neuroinflammation, oxidative stress, and inhibition of key enzymes involved in forming amyloid plaques and other toxic products [284]. Flavonoids exert their neuroprotective effects by preserving neuronal quality and number in critical brain areas, preventing the onset/progression of illnesses that cause a decline in cognitive function.

#### 2.3.3. Stilbenes

The antioxidant and anti-inflammatory characteristics of stilbenoids are primarily responsible for their neuroprotective effects [285,286,287]. Neurodegenerative illnesses like Parkinson’s and Alzheimer’s are linked to oxidative stress and mitochondrial malfunction, which causes neurons to lose function and die [288]. Resveratrol protects neurons from ROS and enhances motor coordination in mice with 1-methyl-4-phenyl-1,2,3,6-tetrahydropyridine (MPTP)-induced parkinsonism by scavenging hydroxyl radicals [289]. Saleh et al. showed that resveratrol could inhibit microglial activation by suppressing NFkB signalling and protect against dopaminergic neurotoxicity caused by LPS [290]. Alzheimer’s disease is marked by the formation of amyloid-beta protein plaques in the hippocampus and cerebral cortex. Amyloid-beta aggregation is necessary for the aetiology of Alzheimer’s disease [291]. Amyloid-beta is also thought to contribute to oxidative damage in neurons by causing lipid peroxidation, protein oxidation, and DNA oxidation [292]. Due to its capacity to decrease amyloid plaques in the brain, resveratrol has therapeutic potential in treating Alzheimer’s disease. Although resveratrol did not reduce amyloid-beta synthesis according to Marambaud et al., it did increase proteasome-dependent amyloid-beta breakdown [293]. As demonstrated in the studies by Yang et al. (2016), resveratrol pretreatment protects against ischemia-reperfusion injury to the rat brain [294]. In resveratrol-treated rats, levels of nuclear factor erythroid 2-related factor (Nrf2) and heme oxygenase-1 (HO-1) were increased, indicating a reduction in oxidative damage during cerebral ischemia [295]. Resveratrol inhibited neuronal death in a rat model of global cerebral ischemia by activating PI3K-Akt signalling and downregulating GSK-3 and cAMP response element-binding protein (CREB) [296]. 

## 3. Conclusions

The control of autophagy is an essential process to maintain cellular homeostasis, thereby increasing survival and suppressing damages during aging. Age-associated declines in health are known to be mediated by disruption of the autophagic equilibrium leading to development of chronic health problems in the elderly, particularly three common illnesses: cancer, DM, and neurodegenerative diseases.

Polyphenols commonly found in our diets could be considered as another natural way to help correct imbalanced autophagy in those chronic diseases and improve overall health status during aging. Several previous reports have confirmed the abilities of different polyphenols in autophagic control, either by activation or inhibition, which results in either cytoprotective or cytotoxic actions depending on the concentrations used and the circumstances of each disease. In this review, more than twenty polyphenolic compounds were discussed in terms of their potential benefits for the treatment of cancer growth and its resistance to standard therapy, DM, and its complications, as well as neurodegenerative diseases, with emphasizing on the underlying molecular mechanisms and signalling pathways related to autophagy. Among them, the stilbene resveratrol holds considerable promise for clinical application as its treatment could promote cell death in many types of cancers, sensitise the resistant cancer cells to therapy, restore insulin secretion, alleviate diabetic complications, as well as protect against neuronal damage through modulation of autophagy.

Natural compounds like polyphenols can be very useful and continue to be of scientific interest due to their multifunctional biological properties and abundant availability in various dietary sources. Several polyphenolic compounds presented in this review, especially resveratrol, deserve to be investigated further in suitable clinical study designs with appropriate doses, to develop effective and promising alternative therapeutics for overall health maintenance as we age.

## Data Availability

Data sharing not applicable.

## References

[1] Manach C., Scalbert A., Morand C., Rémésy C., Jiménez L. (2004). Polyphenols: Food sources and bioavailability. Am. J. Clin. Nutr..

[2] Pandey K.B., Rizvi S.I. (2009). Plant Polyphenols as Dietary Antioxidants in Human Health and Disease. Oxidative Med. Cell. Longev..

[3] Truzzi F., Tibaldi C., Zhang Y., Dinelli G., D′amen E. (2021). An Overview on Dietary Polyphenols and Their Biopharmaceutical Classification System (BCS). Int. J. Mol. Sci..

[4] Del Rio D., Costa L.G., Lean M.E.J., Crozier A. (2010). Polyphenols and health: What compounds are involved?. Nutr. Metab. Cardiovasc. Dis..

[5] Sellappan S., Akoh C.C., Krewer G. (2002). Phenolic Compounds and Antioxidant Capacity of Georgia-Grown Blueberries and Blackberries. J. Agric. Food Chem..

[6] Zuo Y., Wang C., Zhan J. (2002). Separation, Characterization, and Quantitation of Benzoic and Phenolic Antioxidants in American Cranberry Fruit by GC−MS. J. Agric. Food Chem..

[7] Naczk M., Shahidi F. (2006). Phenolics in cereals, fruits and vegetables: Occurrence, extraction and analysis. J. Pharm. Biomed. Anal..

[8] Zamora-Ros R., Rothwell J.A., Scalbert A., Knaze V., Romieu I., Slimani N., Fagherazzi G., Perquier F., Touillaud M., Molina-Montes E. (2013). Dietary intakes and food sources of phenolic acids in the European Prospective Investigation into Cancer and Nutrition (EPIC) study. Br. J. Nutr..

[9] Vareed S.K., Kakarala M., Ruffin M.t., Crowell J.A., Normolle D.P., Djuric Z., Brenner D.E. (2008). Pharmacokinetics of Curcumin Conjugate Metabolites in Healthy Human Subjects. Cancer Epidemiol. Biomark. Prev..

[10] Justesen U., Knuthsen P. (2001). Composition of flavonoids in fresh herbs and calculation of flavonoid intake by use of herbs in traditional Danish dishes. Food Chem..

[11] Tomás-Barberán F.A., Clifford M.N. (2000). Flavanones, chalcones and dihydrochalcones—Nature, occurrence and dietary bur-den. J. Sci. Food Agric..

[12] Han J., Britten M., St-Gelais D., Champagne C.P., Fustier P., Salmieri S., Lacroix M. (2011). Polyphenolic compounds as functional ingredients in cheese. Food Chem..

[13] Spagnuolo C., Russo G.L., Orhan I.E., Habtemariam S., Daglia M., Sureda A., Nabavi S.F., Devi K.P., Loizzo M.R., Tundis R. (2015). Genistein and Cancer: Current Status, Challenges, and Future Directions. Adv. Nutr..

[14] Popa D.-S., Rusu M.E., Shiomi N., Waisundara V. (2017). Isoflavones: Vegetable Sources, Biological Activity, and Analytical Methods for Their Assessment. Superfood and Functional Food—The Development of Superfoods and Their Roles as Medicine.

[15] Sun-Waterhouse D., Zhou J., Wadhwa S.S. (2013). Drinking yoghurts with berry polyphenols added before and after fermentation. Food Control..

[16] Rue E.A., Rush M.D., Van Breemen R.B. (2018). Procyanidins: A comprehensive review encompassing structure elucidation via mass spectrometry. Phytochem. Rev..

[17] Hammerstone J.F., Lazarus S.A., Schmitz H.H. (2000). Procyanidin Content and Variation in Some Commonly Consumed Foods. J. Nutr..

[18] Kondo S., Tsuda K., Muto N., Ueda J.-E. (2002). Antioxidative activity of apple skin or flesh extracts associated with fruit development on selected apple cultivars. Sci. Hortic..

[19] Hertog M.G.L., Hollman P.C.H., Van De Putte B. (1993). Content of potentially anticarcinogenic flavonoids of tea infusions, wines, and fruit juices. J. Agric. Food Chem..

[20] Arts I.C.W., Van De Putte B., Hollman P.C.H. (2000). Catechin Contents of Foods Commonly Consumed in The Netherlands. 1. Fruits, Vegetables, Staple Foods, and Processed Foods. J. Agric. Food Chem..

[21] Cione E., La Torre C., Cannataro R., Caroleo M.C., Plastina P., Gallelli L. (2019). Quercetin, Epigallocatechin Gallate, Curcumin, and Resveratrol: From Dietary Sources to Human MicroRNA Modulation. Molecules.

[22] Chu Q., O’Dwye M., Zeece M.G. (1998). Direct Analysis of Resveratrol in Wine by Micellar Electrokinetic Capillary Electrophoresis. J. Agric. Food Chem..

[23] Sudhakar P., Jacomin A.-C., Hautefort I., Samavedam S., Fatemian K., Ari E., Gul L., Demeter A., Jones E., Korcsmaros T. (2019). Targeted interplay between bacterial pathogens and host autophagy. Autophagy.

[24] Brimson J.M., Prasanth M.I., Malar D.S., Brimson S., Thitilertdecha P., Tencomnao T. (2021). Drugs that offer the potential to reduce hospitalization and mortality from SARS-CoV-2 infection: The possible role of the Sigma-1 receptor and autophagy. Expert Opin. Ther. Targets.

[25] Prasanth M.I., Malar D., Tencomnao T., Brimson J. (2021). The emerging role of the sigma-1 receptor in autophagy: Hand-in-hand targets for the treatment of Alzheimer’s. Expert Opin. Ther. Targets.

[26] Fu Y., Chang H., Peng X., Bai Q., Yi L., Zhou Y., Zhu J., Mi M. (2014). Resveratrol Inhibits Breast Cancer Stem-Like Cells and Induces Autophagy via Suppressing Wnt/β-Catenin Signaling Pathway. PLoS ONE.

[27] Sarparanta J., Garcia-Macia M., Singh R. (2017). Autophagy and Mitochondria in Obesity and Type 2 Diabetes. Curr. Diabetes Rev..

[28] Takeshige K., Baba M., Tsuboi S., Noda T., Ohsumi Y. (1992). Autophagy in yeast demonstrated with proteinase-deficient mutants and conditions for its induction. J. Cell Biol..

[29] Klionsky D.J., Cregg J.M., Dunn W.A., Emr S.D., Sakai Y., Sandoval I.V., Sibirny A., Subramani S., Thumm M., Veenhuis M. (2003). A Unified Nomenclature for Yeast Autophagy-Related Genes. Dev. Cell.

[30] Lamb C., Yoshimori T., Tooze S. (2013). The autophagosome: Origins unknown, biogenesis complex. Nat. Rev. Mol. Cell Biol..

[31] Russell R.C., Yuan H.-X., Guan K.-L. (2014). Autophagy regulation by nutrient signaling. Cell Res..

[32] Hay N., Sonenberg N. (2004). Upstream and downstream of mTOR. Genome Res. Dev..

[33] Jeon S.-M. (2016). Regulation and function of AMPK in physiology and diseases. Exp. Mol. Med..

[34] Kuroyanagi H., Yan J., Seki N., Yamanouchi Y., Suzukia Y., Takano T., Muramatsu M., Shirasawa T. (1998). Human ULK1, a Novel Serine/Threonine Kinase Related to UNC-51 Kinase ofCaenorhabditis elegans:cDNA Cloning, Expression, and Chromosomal Assignment. Genomics.

[35] Lin M.G., Hurley J.H. (2016). Structure and function of the ULK1 complex in autophagy. Curr. Opin. Cell Biol..

[36] Russell R.C., Tian Y., Yuan H., Park H.W., Chang Y.-Y., Kim J., Kim H., Neufeld T.P., Dillin A., Guan K.-L. (2013). ULK1 induces autophagy by phosphorylating Beclin-1 and activating VPS34 lipid kinase. Nature.

[37] Lazarus M.B., Novotny C.J., Shokat K.M. (2015). Structure of the Human Autophagy Initiating Kinase ULK1 in Complex with Potent Inhibitors. ACS Chem. Biol..

[38] Axe E.L., Walker S.A., Manifava M., Chandra P., Roderick H.L., Habermann A., Griffiths G., Ktistakis N.T. (2008). Autophagosome formation from membrane compartments enriched in phosphatidylinositol 3-phosphate and dynamically connected to the endoplasmic reticulum. J. Cell Biol..

[39] Biazik J., Ylä-Anttila P., Vihinen H., Jokitalo E., Eskelinen E.-L. (2015). Ultrastructural relationship of the phagophore with surrounding organelles. Autophagy.

[40] Kruppa A.J., Kendrick-Jones J., Buss F. (2016). Myosins, Actin and Autophagy. Traffic.

[41] Mizushima N., Ohsumi Y., Yoshimori T. (2002). Autophagosome Formation in Mammalian Cells. Cell Struct. Funct..

[42] Yoshii S.R., Mizushima N. (2017). Monitoring and Measuring Autophagy. Int. J. Mol. Sci..

[43] Klionsky D.J., Abdel-Aziz A.K., Abdelfatah S., Abdellatif M., Abdoli A., Abel S., Abeliovich H., Abildgaard M.H., Abudu Y.P., Acevedo-Arozena A. (2021). Guidelines for the use and interpretation of assays for monitoring autophagy. Autophagy.

[44] Zhao Y.G., Codogno P., Zhang H. (2021). Machinery, regulation and pathophysiological implications of autophagosome matu-ration. Nat. Rev. Mol. Cell Biol..

[45] Degenhardt K., Mathew R., Beaudoin B., Bray K., Anderson D., Chen G., Mukherjee C., Shi Y., Gélinas C., Fan Y. (2006). Autophagy promotes tumor cell survival and restricts necrosis, inflammation, and tumorigenesis. Cancer Cell.

[46] Pavlides S., Vera I., Gandara R., Sneddon S., Pestell R.G., Mercier I., Martinez-Outschoorn U.E., Whitaker-Menezes D., Howell A., Sotgia F. (2012). Warburg Meets Autophagy: Cancer-Associated Fibroblasts Accelerate Tumor Growth and Metastasis via Oxidative Stress, Mitophagy, and Aerobic Glycolysis. Antioxid. Redox Signal..

[47] Shen Y., Li D.-D., Wang L.-L., Deng R., Zhu X.-F. (2008). Decreased expression of autophagy-related proteins in malignant epithelial ovarian cancer. Autophagy.

[48] Qu X., Yu J., Bhagat G., Furuya N., Hibshoosh H., Troxel A., Rosen J., Eskelinen E.-L., Mizushima N., Ohsumi Y. (2003). Promotion of tumorigenesis by heterozygous disruption of the beclin 1 autophagy gene. J. Clin. Investig..

[49] Liang X.H., Jackson S., Seaman M., Brown K., Kempkes B., Hibshoosh H., Levine B. (1999). Induction of autophagy and inhibition of tumorigenesis by beclin 1. Nature.

[50] Takahashi Y., Coppola D., Matsushita N., Cualing H.D., Sun M., Sato Y., Liang C., Jung J.U., Cheng J.Q., Mul J.J. (2007). Bif-1 interacts with Beclin 1 through UVRAG and regulates autophagy and tumorigenesis. Nature.

[51] He S., Zhao Z., Yang Y., O’Connell D., Zhang X., Oh S., Ma B., Lee J.-H., Zhang T., Varghese B. (2015). Truncating mutation in the autophagy gene UVRAG confers oncogenic properties and chemosensitivity in colorectal cancers. Nat. Commun..

[52] Kim M.S., Jeong E.G., Ahn C.H., Kim S.S., Lee S.H., Yoo N.J. (2008). Frameshift mutation of UVRAG, an autophagy-related gene, in gastric carcinomas with microsatellite instability. Hum. Pathol..

[53] Valente G., Morani F., Nicotra G., Fusco N., Peracchio C., Titone R., Alabiso O., Arisio R., Katsaros D., Benedetto C. (2014). Expression and Clinical Significance of the Autophagy Proteins BECLIN 1 and LC3 in Ovarian Cancer. BioMed Res. Int..

[54] Takamura A., Komatsu M., Hara T., Sakamoto A., Kishi C., Waguri S., Eishi Y., Hino O., Tanaka K., Mizushima N. (2011). Autophagy-deficient mice develop multiple liver tumors. Genes Dev..

[55] Yang A., RajeshKumar N., Wang X., Yabuuchi S., Alexander B.M., Chu G.C., Von Hoff D.D., Maitra A., Kimmelman A.C. (2014). Autophagy Is Critical for Pancreatic Tumor Growth and Progression in Tumors with p53 Alterations. Cancer Discov..

[56] Strohecker A.M., Guo J.Y., Karsli-Uzunbas G., Price S.M., Chen G.J., Mathew R., McMahon M., White E. (2013). Autophagy Sustains Mitochondrial Glutamine Metabolism and Growth of BrafV600E–Driven Lung Tumors. Cancer Discov..

[57] Sou Y.-S., Waguri S., Iwata J.-I., Ueno T., Fujimura T., Hara T., Sawada N., Yamada A., Mizushima N., Uchiyama Y. (2008). The Atg8 Conjugation System Is Indispensable for Proper Development of Autophagic Isolation Membranes in Mice. Mol. Biol. Cell.

[58] Levine B., Kroemer G. (2019). Biological Functions of Autophagy Genes: A Disease Perspective. Cell.

[59] Lawson K.A., Sousa C.M., Zhang X., Kim E., Akthar R., Caumanns J.J., Yao Y., Mikolajewicz N., Ross C., Brown K.R. (2020). Functional genomic landscape of cancer-intrinsic evasion of killing by T cells. Nature.

[60] Katheder N.S., Khezri R., Ofarrell F., Schultz S.W., Jain A., Rahman M.M., Schink K.O., Theodossiou T.A., Johansen T., Juhasz G. (2017). Microenvironmental autophagy promotes tumour growth. Nature.

[61] Guo J.Y., Chen H.-Y., Mathew R., Fan J., Strohecker A.M., Karsli-Uzunbas G., Kamphorst J.J., Chen G., Lemons J.M., Karantza V. (2011). Activated Ras requires autophagy to maintain oxidative metabolism and tumorigenesis. Genes Dev..

[62] Poillet-Perez L., Xie X., Zhan L., Yang Y., Sharp D.W., Hu Z.S., Su X., Maganti A., Jiang C., Lu W. (2018). Autophagy maintains tumour growth through circulating arginine. Nature.

[63] Xie X., Koh J.Y., Price S., White E., Mehnert J.M. (2015). Atg7 Overcomes Senescence and Promotes Growth of BrafV600E-Driven Melanoma. Cancer Discov..

[64] Kim H.-S., Quon M.J., Kim J.-A. (2014). New insights into the mechanisms of polyphenols beyond antioxidant properties; lessons from the green tea polyphenol, epigallocatechin 3-gallate. Redox Biol..

[65] Harborne J., A Williams C. (2000). Advances in flavonoid research since 1992. Phytochemistry.

[66] Yoo H.S., Won S.B., Kwon Y.H. (2021). Luteolin Induces Apoptosis and Autophagy in HCT116 Colon Cancer Cells via p53-Dependent Pathway. Nutr. Cancer.

[67] Kang K.A., Piao M.J., Hyun Y.J., Zhen A.X., Cho S.J., Ahn M.J., Yi J.M., Hyun J.W. (2019). Luteolin promotes apoptotic cell death via upregulation of Nrf2 expression by DNA demethylase and the interaction of Nrf2 with p53 in human colon cancer cells. Exp. Mol. Med..

[68] Zuo Q., Wu R., Xiao X., Yang C., Yang Y., Wang C., Lin L., Kong A.N. (2018). The dietary flavone luteolin epigenetically activates the Nrf2 pathway and blocks cell transformation in human colorectal cancer HCT116 cells. J. Cell. Biochem..

[69] Lee Y., Kwon Y.H. (2018). Regulation of apoptosis and autophagy by luteolin in human hepatocellular cancer Hep3B cells. Biochem. Biophys. Res. Commun..

[70] Nazim U.M., Park S.Y. (2018). Luteolin sensitizes human liver cancer cells to TRAIL-induced apoptosis via autophagy and JNK-mediated death receptor 5 upregulation. Int. J. Oncol..

[71] Cao Z., Zhang H., Cai X., Fang W., Chai D., Wen Y., Chen H., Chu F., Zhang Y. (2017). Luteolin Promotes Cell Apoptosis by Inducing Autophagy in Hepatocellular Carcinoma. Cell. Physiol. Biochem..

[72] Park S.-H., Park H.S., Lee J.H., Chi G.Y., Kim G.-Y., Moon S.-K., Chang Y.-C., Hyun J.W., Kim W.-J., Choi Y.H. (2013). Induction of endoplasmic reticulum stress-mediated apoptosis and non-canonical autophagy by luteolin in NCI-H460 lung carcinoma cells. Food Chem. Toxicol..

[73] Zhang M., Wang R., Tian J., Song M., Zhao R., Liu K., Zhu F., Shim J.H., Dong Z., Lee M.H. (2021). Targeting LIMK1 with luteolin inhibits the growth of lung cancer in vitro and in vivo. J. Cell. Mol. Med..

[74] Hamill S., Lou H.J., Turk B.E., Boggon T.J. (2016). Structural Basis for Noncanonical Substrate Recognition of Cofilin/ADF Proteins by LIM Kinases. Mol. Cell.

[75] Verschooten L., Barrette K., Van Kelst S., Romero N.R., Proby C., De Vos R., Agostinis P., Garmyn M. (2012). Autophagy Inhibitor Chloroquine Enhanced the Cell Death Inducing Effect of the Flavonoid Luteolin in Metastatic Squamous Cell Carcinoma Cells. PLoS ONE.

[76] Chakrabarti M., Ray S.K. (2016). Anti-tumor activities of luteolin and silibinin in glioblastoma cells: Overexpression of miR-7-1-3p augmented luteolin and silibinin to inhibit autophagy and induce apoptosis in glioblastoma in vivo. Apoptosis.

[77] Liu Q., Zhu D., Hao B., Zhang Z., Tian Y. (2018). Luteolin promotes the sensitivity of cisplatin in ovarian cancer by decreasing PRPA1-medicated autophagy. Cell. Mol. Biol..

[78] Chen Z.-T., Zhao W., Qu S., Li L., Lu X.-D., Su F., Liang Z.-G., Guo S.-Y., Zhu X.-D. (2015). PARP-1 promotes autophagy via the AMPK/mTOR pathway in CNE-2 human nasopharyngeal carcinoma cells following ionizing radiation, while inhibition of autophagy contributes to the radiation sensitization of CNE-2 cells. Mol. Med. Rep..

[79] Brimson J.M., Prasanth M.I., Malar D.S., Sharika R., Sivamaruthi B.S., Kesika P., Chaiyasut C., Tencomnao T., Prasansuklab A. (2021). Role of Herbal Teas in Regulating Cellular Homeostasis and Autophagy and Their Implications in Regulating Overall Health. Nutrients.

[80] Malar D.S., Prasanth M.I., Brimson J.M., Sharika R., Sivamaruthi B.S., Chaiyasut C., Tencomnao T. (2020). Neuroprotective Properties of Green Tea (*Camellia sinensis*) in Parkinson’s Disease: A Review. Molecules.

[81] Prasanth M.I., Sivamaruthi B.S., Chaiyasut C., Tencomnao T. (2019). A Review of the Role of Green Tea (*Camellia sinensis*) in Antiphotoaging, Stress Resistance, Neuroprotection, and Autophagy. Nutrients.

[82] Sulistiyani E., Brimson J.M., Chansaenroj A., Sariya L., Urkasemsin G., Oonsiri S., Tencomnao T., Vacharaksa A., Chaisuparat R., Ferreira J.N. (2021). Epigallocatechin-3-Gallate Protects Pro-Acinar Epithelia Against Salivary Gland Radiation Injury. Int. J. Mol. Sci..

[83] Zhang Z., Zhang S., Yang J., Yi P., Xu P., Yi M., Peng W. (2020). Integrated transcriptomic and metabolomic analyses to characterize the anti-cancer effects of (−)-epigallocatechin-3-gallate in human colon cancer cells. Toxicol. Appl. Pharmacol..

[84] Kim S.-W., Moon J.-H., Park S.-Y. (2016). Activation of autophagic flux by epigallocatechin gallate mitigates TRAIL-induced tumor cell apoptosis via down-regulation of death receptors. Oncotarget.

[85] Wu W., Dong J., Gou H., Geng R., Yang X., Chen D., Xiang B., Zhang Z., Ren S., Chen L. (2021). EGCG synergizes the therapeutic effect of irinotecan through enhanced DNA damage in human colorectal cancer cells. J. Cell. Mol. Med..

[86] Alexander A., Kim J., Walker C.L. (2010). ATM engages the TSC2/mTORC1 signaling node to regulate autophagy. Autophagy.

[87] Hu F., Wei F., Wang Y., Wu B., Fang Y., Xiong B. (2015). EGCG synergizes the therapeutic effect of cisplatin and oxaliplatin through autophagic pathway in human colorectal cancer cells. J. Pharmacol. Sci..

[88] Enkhbat T., Nishi M., Yoshikawa K., Jun H., Tokunaga T., Takasu C., Kashihara H., Ishikawa D., Tominaga M., Shimada M. (2018). Epigallocatechin-3-gallate Enhances Radiation Sensitivity in Colorectal Cancer Cells Through Nrf2 Activation and Autophagy. Anticancer Res..

[89] Yin Z., Li J., Kang L., Liu X., Luo J., Zhang L., Li Y., Cai J. (2021). Epigallocatechin-3-gallate induces autophagy-related apoptosis associated with LC3B II and Beclin expression of bladder cancer cells. J. Food Biochem..

[90] Meng J., Chang C., Chen Y., Bi F., Ji C., Liu W. (2019). EGCG overcomes gefitinib resistance by inhibiting autophagy and augmenting cell death through targeting ERK phosphorylation in NSCLC. OncoTargets Ther..

[91] Wei R., Mao L., Xu P., Zheng X., Hackman R.M., Mackenzie G.G., Wang Y. (2018). Suppressing glucose metabolism with epigallocatechin-3-gallate (EGCG) reduces breast cancer cell growth in preclinical models. Food Funct..

[92] Del Rey M.J., Valín Á., Usategui A., Garcia C.M., Sánchez-Aragó M., Cuezva J.M., Galindo M., Bravo B., Cañete J.D., Blanco F.J. (2017). Hif-1α Knockdown Reduces Glycolytic Metabolism and Induces Cell Death of Human Synovial Fibroblasts Under Normoxic Conditions. Sci. Rep..

[93] Zhao L., Liu S., Xu J., Li W., Duan G., Wang H., Yang H., Yang Z., Zhou R. (2017). A new molecular mechanism underlying the EGCG-mediated autophagic modulation of AFP in HepG2 cells. Cell Death Dis..

[94] Yuan C.-H., Horng C.-T., Lee C.-F., Chiang N.-N., Tsai F.-J., Lu C.-C., Chiang J.-H., Hsu Y.-M., Yang J.-S., Chen F.-A. (2017). Epigallocatechin gallate sensitizes cisplatin-resistant oral cancer CAR cell apoptosis and autophagy through stimulating AKT/STAT3 pathway and suppressing multidrug resistance 1 signaling. Environ. Toxicol..

[95] Modernelli A., Naponelli V., Troglio M.G., Bonacini M., Ramazzina I., Bettuzzi S., Rizzi F. (2015). EGCG antagonizes Bortezomib cytotoxicity in prostate cancer cells by an autophagic mechanism. Sci. Rep..

[96] Wang W., Chen D., Zhu K. (2018). SOX2OT variant 7 contributes to the synergistic interaction between EGCG and Doxorubicin to kill osteosarcoma via autophagy and stemness inhibition. J. Exp. Clin. Cancer Res..

[97] Chen L., Ye H.-L., Zhang G., Yao W.-M., Chen X.-Z., Zhang F.-C., Liang G. (2014). Autophagy Inhibition Contributes to the Synergistic Interaction between EGCG and Doxorubicin to Kill the Hepatoma Hep3B Cells. PLoS ONE.

[98] Chen X., Tong R., Liu B., Liu H., Feng X., Ding S., Lei Q., Tang G., Wu J., Fang W. (2020). Duo of (–)-epigallocatechin-3-gallate and doxorubicin loaded by polydopamine coating ZIF-8 in the regulation of autophagy for chemo-photothermal synergistic therapy. Biomater. Sci..

[99] Cucciolla V., Borriello A., Oliva A., Galletti P., Zappia V., Della Ragione F. (2007). Resveratrol: From Basic Science to the Clinic. Cell Cycle.

[100] Zahedi H.S., Jazayeri S., Ghiasvand R., Djalali M., Eshraghian M.R. (2013). Effects of Polygonum Cuspidatum Containing Resveratrol on Inflammation in Male Professional Basketball Players. Int. J. Prev. Med..

[101] Lai T.N.H., André C.M., Chirinos R., Nguyen T.B.T., Larondelle Y., Rogez H. (2014). Optimisation of extraction of piceatannol from Rhodomyrtus tomentosa seeds using response surface methodology. Sep. Purif. Technol..

[102] Trinh T.A., Lee D., Park S., Kim S.H., Park J.G., Kim J.H., Kang K.S. (2019). Stilbenes contribute to the anticancer effects of Rheum undulatum L. through activation of apoptosis. Oncol. Lett..

[103] Tsuruga T., Chun Y.-T., Ebizuka Y., Sankawa U. (1991). Biologically Active constituents of Melaleuca leucadendron: Inhibitors of Induced Histamine Release from Rat Mast Cells. Chem. Pharm. Bull..

[104] Zheng J., Wei S., Xiao T., Li G. (2021). LC3B/p62-mediated mitophagy protects A549 cells from resveratrol-induced apoptosis. Life Sci..

[105] Wang J., Li J., Cao N., Li Z., Han J., Li L. (2018). Resveratrol, an activator of SIRT1, induces protective autophagy in non-small-cell lung cancer via inhibiting Akt/mTOR and activating p38-MAPK. OncoTargets Ther..

[106] Rasheduzzaman M., Jeong J.-K., Park S.-Y. (2018). Resveratrol sensitizes lung cancer cell to TRAIL by p53 independent and suppression of Akt/NF-κB signaling. Life Sci..

[107] Reya T., Clevers H. (2005). Wnt signalling in stem cells and cancer. Nature.

[108] Gao C., Cao W., Bao L., Zuo W., Xie G., Cai T., Fu W., Zhang J., Wu W., Zhang X. (2010). Autophagy negatively regulates Wnt signalling by promoting Dishevelled degradation. Nature.

[109] Petherick K.J., Williams A.C., Lane J.D., Ordóñez-Morán P., Huelsken J., Collard T.J., Smartt H.J., Batson J., Malik K., Paraskeva C. (2013). Autolysosomal β-catenin degradation regulates Wnt-autophagy-p62 crosstalk. EMBO J..

[110] Vargas J.E., Puga R., Lenz G., Trindade C., Filippi-Chiela E. (2020). Cellular Mechanisms Triggered by the Cotreatment of Resveratrol and Doxorubicin in Breast Cancer: A Translational In Vitro–In Silico Model. Oxidative Med. Cell. Longev..

[111] Kumar B., Iqbal M.A., Singh R.K., Bamezai R.N. (2015). Resveratrol inhibits TIGAR to promote ROS induced apoptosis and autophagy. Biochimie.

[112] Xie J.-M., Li B., Yu H.-P., Gao Q.-G., Li W., Wu H.-R., Qin Z.-H. (2014). TIGAR Has a Dual Role in Cancer Cell Survival through Regulating Apoptosis and Autophagy. Cancer Res..

[113] Rodríguez-Enríquez S., Pacheco-Velázquez S.C., Marín-Hernández Á., Gallardo-Pérez J.C., Robledo-Cadena D.X., Hernández-Reséndiz I., García-García J.D., Belmont-Díaz J., López-Marure R., Hernández-Esquivel L. (2019). Resveratrol inhibits cancer cell proliferation by impairing oxidative phosphorylation and inducing oxidative stress. Toxicol. Appl. Pharmacol..

[114] Wang H., Peng Y., Wang J., Gu A., Li Q., Mao D., Guo L. (2019). Effect of autophagy on the resveratrol-induced apoptosis of ovarian cancer SKOV3 cells. J. Cell. Biochem..

[115] Ferraresi A., Phadngam S., Morani F., Galetto A., Alabiso O., Chiorino G., Isidoro C. (2017). Resveratrol inhibits IL-6-induced ovarian cancer cell migration through epigenetic up-regulation of autophagy. Mol. Carcinog..

[116] Lu Z., Luo R.Z., Lu Y., Zhang X., Yu Q., Khare S., Kondo S., Kondo Y., Yu Y., Mills G.B. (2008). The tumor suppressor gene ARHI regulates autophagy and tumor dormancy in human ovarian cancer cells. J. Clin. Investig..

[117] Kueck A., Opipari A.W., Griffith K.A., Tan L., Choi M., Huang J., Wahl H., Liu J.R. (2007). Resveratrol inhibits glucose metabolism in human ovarian cancer cells. Gynecol. Oncol..

[118] Hsu K.-F., Wu C.-L., Huang S.-C., Wu C.-M., Hsiao J.-R., Yo Y.-T., Chen Y.-H., Shiau A.-L., Chou C.-Y. (2009). Cathepsin L mediates resveratrol-induced autophagy and apoptotic cell death in cervical cancer cells. Autophagy.

[119] Chang N.C., Nguyen M., Germain M., Shore G.C. (2010). Antagonism of Beclin 1-dependent autophagy by BCL-2 at the endoplasmic reticulum requires NAF-1. EMBO J..

[120] Cheng L., Yan B., Chen K., Jiang Z., Zhou C., Cao J., Qian W., Li J., Sun L., Ma J. (2018). Resveratrol-Induced Downregulation of NAF-1 Enhances the Sensitivity of Pancreatic Cancer Cells to Gemcitabine via the ROS/Nrf2 Signaling Pathways. Oxidative Med. Cell. Longev..

[121] Qin T., Cheng L., Xiao Y., Qian W., Li J., Wu Z., Wang Z., Xu Q., Duan W., Wong L. (2020). NAF-1 Inhibition by Resveratrol Suppresses Cancer Stem Cell-Like Properties and the Invasion of Pancreatic Cancer. Front. Oncol..

[122] Zeng Y., Li F.-D., Shi C.-W., Du J.-L., Xue Y.-J., Liu X.-Y., Cao X., Wei N. (2020). Mechanism and therapeutic prospect of resveratrol combined with TRAIL in the treatment of renal cell carcinoma. Cancer Gene Ther..

[123] Thongchot S., Ferraresi A., Vidoni C., Loilome W., Yongvanit P., Namwat N., Isidoro C. (2018). Resveratrol interrupts the pro-invasive communication between cancer associated fibroblasts and cholangiocarcinoma cells. Cancer Lett..

[124] Zhu H., Ding J., Jiao M., Wu J., Liu T., Liang J., Tang Q. (2017). Resveratrol attenuates bone cancer pain through regulating the expression levels of ASIC3 and activating cell autophagy. Acta Biochim. Biophys. Sin..

[125] Chang C.-H., Lee C.-Y., Lu C.-C., Tsai F.-J., Hsu Y.-M., Tsao J.-W., Juan Y.-N., Chiu H.-Y., Yang J.-S., Wang C.-C. (2017). Resveratrol-induced autophagy and apoptosis in cisplatin-resistant human oral cancer CAR cells: A key role of AMPK and Akt/mTOR signaling. Int. J. Oncol..

[126] Zhang B., Yin X., Sui S. (2018). Resveratrol inhibited the progression of human hepatocellular carcinoma by inducing autophagy via regulating p53 and the phosphoinositide 3-kinase/protein kinase B pathway. Oncol. Rep..

[127] Signorelli P., Munoz-Olaya J.M., Gagliostro V., Casas J., Ghidoni R., Fabrias G. (2009). Dihydroceramide intracellular increase in response to resveratrol treatment mediates autophagy in gastric cancer cells. Cancer Lett..

[128] Wang M., Yu T., Zhu C., Sun H., Qiu Y., Zhu X., Li J. (2014). Resveratrol Triggers Protective Autophagy Through the Ceramide/Akt/mTOR Pathway in Melanoma B16 Cells. Nutr. Cancer.

[129] Fan Y., Chiu J.-F., Liu J., Deng Y., Xu C., Zhang J., Li G. (2018). Resveratrol induces autophagy-dependent apoptosis in HL-60 cells. BMC Cancer.

[130] Trincheri N.F., Follo C., Nicotra G., Peracchio C., Castino R., Isidoro C. (2008). Resveratrol-induced apoptosis depends on the lipid kinase activity of Vps34 and on the formation of autophagolysosomes. Carcinogenesis.

[131] Perrone D., Ardito F., Giannatempo G., Dioguardi M., Troiano G., Lo Russo L., De Lillo A., Laino L., Lo Muzio L. (2015). Biological and therapeutic activities, and anticancer properties of curcumin. Exp. Ther. Med..

[132] Gunarta I.K., Yuliana D., Erdenebaatar P., Kishi Y., Boldbaatar J., Suzuki R., Odongoo R., Davaakhuu G., Hohjoh H., Yoshioka K. (2021). c-Jun NH2-terminal kinase (JNK)/stress-activated protein kinase-associated protein 1 (JSAP1) attenuates curcumin-induced cell death differently from its family member, JNK-associated leucine zipper protein (JLP). Drug Discov. Ther..

[133] Khaket T.P., Singh M.P., Khan I., Kang S.C. (2020). In vitro and in vivo studies on potentiation of curcumin-induced lysosomal-dependent apoptosis upon silencing of cathepsin C in colorectal cancer cells. Pharmacol. Res..

[134] Zhang J., Wang J., Xu J., Lu Y., Jiang J., Wang L., Shen H.-M., Xia D. (2016). Curcumin targets the TFEB-lysosome pathway for induction of autophagy. Oncotarget.

[135] Zhu J., Zhao B., Xiong P., Wang C., Zhang J., Tian X., Huang Y. (2018). Curcumin Induces Autophagy via Inhibition of Yes-Associated Protein (YAP) in Human Colon Cancer Cells. Med. Sci. Monit..

[136] Sudol M. (1994). Yes-associated protein (YAP65) is a proline-rich phosphoprotein that binds to the SH3 domain of the Yes proto-oncogene product. Oncogene.

[137] Liu Z., Zeng W., Wang S., Zhao X., Guo Y., Yu P., Yin X., Liu C., Huang T. (2017). A potential role for the Hippo pathway protein, YAP, in controlling proliferation, cell cycle progression, and autophagy in BCPAP and KI thyroid papillary carci-noma cells. Am. J. Transl. Res..

[138] Kaur H., Moreau R. (2021). Curcumin represses mTORC1 signaling in Caco-2 cells by a two-sided mechanism involving the loss of IRS-1 and activation of AMPK. Cell. Signal..

[139] Mao X., Zhang X., Zheng X., Chen Y., Xuan Z., Huang P. (2021). Curcumin suppresses LGR5(+) colorectal cancer stem cells by inducing autophagy and via repressing TFAP2A-mediated ECM pathway. J. Nat. Med..

[140] Huang H.-X., Yang G., Yang Y., Yan J., Tang X.-Y., Pan Q. (2020). TFAP2A is a novel regulator that modulates ferroptosis in gallbladder carcinoma cells via the Nrf2 signalling axis. Eur. Rev. Med. Pharmacol. Sci..

[141] Zhou Y., Shen Y., Chen C., Sui X., Yang J., Wang L., Zhou J. (2019). The crosstalk between autophagy and ferroptosis: What can we learn to target drug resistance in cancer?. Cancer Biol. Med..

[142] Tang X., Ding H., Liang M., Chen X., Yan Y., Wan N., Chen Q., Zhang J., Cao J. (2021). Curcumin induces ferroptosis in non-small-cell lung cancer via activating autophagy. Thorac. Cancer.

[143] Chen P., Huang H.-P., Wang Y., Jin J., Long W.-G., Chen K., Zhao X.-H., Chen C.-G., Li J. (2019). Curcumin overcome primary gefitinib resistance in non-small-cell lung cancer cells through inducing autophagy-related cell death. J. Exp. Clin. Cancer Res..

[144] Lee M., Kim K.-S., Fukushi A., Kim D.-H., Kim C.-H., Lee Y.-C. (2018). Transcriptional Activation of Human GD3 Synthase (hST8Sia I) Gene in Curcumin-Induced Autophagy in A549 Human Lung Carcinoma Cells. Int. J. Mol. Sci..

[145] Matarrese P., Garofalo T., Manganelli V., Gambardella L., Marconi M., Grasso M., Tinari A., Misasi R., Malorni W., Sorice M. (2014). Evidence for the involvement of GD3 ganglioside in autophagosome formation and maturation. Autophagy.

[146] Wang T., Wu X., Al Rudaisat M., Song Y., Cheng H. (2020). Curcumin induces G2/M arrest and triggers autophagy, ROS generation and cell senescence in cervical cancer cells. J. Cancer.

[147] Liu L.-D., Pang Y.-X., Zhao X.-R., Li R., Jin C.-J., Xue J., Dong R.-Y., Liu P.-S. (2019). Curcumin induces apoptotic cell death and protective autophagy by inhibiting AKT/mTOR/p70S6K pathway in human ovarian cancer cells. Arch. Gynecol. Obstet..

[148] Hu P., Ke C., Guo X., Ren P., Tong Y., Luo S., He Y., Wei Z., Cheng B., Li R. (2019). Both glypican-3/Wnt/β-catenin signaling pathway and autophagy contributed to the inhibitory effect of curcumin on hepatocellular carcinoma. Dig. Liver Dis..

[149] Capurro M., Xiang Y.-Y., Lobe C., Filmus J. (2005). Glypican-3 Promotes the Growth of Hepatocellular Carcinoma by Stimulating Canonical Wnt Signaling. Cancer Res..

[150] Miao H.-L., Pan Z.-J., Lei C.-J., Wen J.-Y., Li M.-Y., Liu Z.-K., Qiu Z.-D., Lin M.-Z., Chen N.-P., Chen M. (2013). Knockdown of GPC3 inhibits the proliferation of Huh7 hepatocellular carcinoma cells through down-regulation of YAP. J. Cell. Biochem..

[151] Deng L., Wu X., Zhu X., Yu Z., Liu Z., Wang J., Zheng Y. (2021). Combination effect of curcumin with docetaxel on the PI3K/AKT/mTOR pathway to induce autophagy and apoptosis in esophageal squamous cell carcinoma. Am. J. Transl. Res..

[152] Liu J., Li M., Wang Y., Luo J. (2017). Curcumin sensitizes prostate cancer cells to radiation partly via epigenetic activation of miR-143 and miR-143 mediated autophagy inhibition. J. Drug Target..

[153] Fan X., Chen X., Deng W., Zhong G., Cai Q., Lin T. (2013). Up-regulated microRNA-143 in cancer stem cells differentiation promotes prostate cancer cells metastasis by modulating FNDC3B expression. BMC Cancer.

[154] Guo H., Ding H., Tang X., Liang M., Li S., Zhang J., Cao J. (2021). Quercetin induces pro-apoptotic autophagy via SIRT1 / AMPK signaling pathway in human lung cancer cell lines A549 and H1299 in vitro. Thorac. Cancer.

[155] Moon J.-H., Eo S.K., Lee J.H., Park S.-Y. (2015). Quercetin-induced autophagy flux enhances TRAIL-mediated tumor cell death. Oncol. Rep..

[156] Wang Z.X., Ma J., Li X.Y., Wu Y., Shi H.M., Chen Y., Lu G.D., Shen H., Lu G., Zhou J. (2021). Quercetin induces p53-independent cancer cell death through lysosome activation by the transcription factor EB and Reactive Oxygen Species-dependent ferroptosis. Br. J. Pharmacol..

[157] Wu L., Li J., Liu T., Li S., Feng J., Yu Q., Zhang J., Chen J., Zhou Y., Ji J. (2019). Quercetin shows anti-tumor effect in hepatocellular carcinoma LM3 cells by abrogating JAK2/STAT3 signaling pathway. Cancer Med..

[158] Hyun J.J., Lee H.S., Keum B., Seo Y.S., Jeen Y.T., Chun H.J., Um S.H., Kim C.D. (2013). Expression of Heat Shock Protein 70 Modulates the Chemoresponsiveness of Pancreatic Cancer. Gut Liver.

[159] Dokladny K., Myers O.B., Moseley P.L. (2015). Heat shock response and autophagy—cooperation and control. Autophagy.

[160] Aghdassi A., Phillips P., Dudeja V., Dhaulakhandi D., Sharif R., Dawra R., Lerch M.M., Saluja A. (2007). Heat Shock Protein 70 Increases Tumorigenicity and Inhibits Apoptosis in Pancreatic Adenocarcinoma. Cancer Res..

[161] Lan C.-Y., Chen S.-Y., Kuo C.-W., Lu C.-C., Yen G.-C. (2019). Quercetin facilitates cell death and chemosensitivity through RAGE/PI3K/AKT/mTOR axis in human pancreatic cancer cells. J. Food Drug Anal..

[162] Jia L., Huang S., Yin X., Zan Y., Guo Y., Han L. (2018). Quercetin suppresses the mobility of breast cancer by suppressing glycolysis through Akt-mTOR pathway mediated autophagy induction. Life Sci..

[163] Liu Y., Gong W., Yang Z.Y., Zhou X.S., Gong C., Zhang T.R., Wei X., Ma D., Ye F., Gao Q.L. (2017). Quercetin induces protective autophagy and apoptosis through ER stress via the p-STAT3/Bcl-2 axis in ovarian cancer. Apoptosis.

[164] Jang E., Kim I.Y., Kim H., Lee D.M., Seo D.Y., Lee J.A., Choi K.S., Kim E. (2020). Quercetin and chloroquine synergistically kill glioma cells by inducing organelle stress and disrupting Ca^2+^ homeostasis. Biochem. Pharmacol..

[165] Wu B., Zeng W., Ouyang W., Xu Q., Chen J., Wang B., Zhang X. (2020). Quercetin induced NUPR1-dependent autophagic cell death by disturbing reactive oxygen species homeostasis in osteosarcoma cells. J. Clin. Biochem. Nutr..

[166] Mu Y., Yan X., Li D., Zhao D., Wang L., Wang X., Gao D., Yang J., Zhang H., Li Y. (2018). NUPR1 maintains autolysosomal efflux by activating SNAP25 transcription in cancer cells. Autophagy.

[167] Hamidi T., Cano C.E., Grasso D., Garcia M.N., Sandi M.J., Calvo E.L., Dagorn J.-C., Lomberk G., Urrutia R., Goruppi S. (2012). Nupr1-Aurora Kinase A Pathway Provides Protection against Metabolic Stress-Mediated Autophagic-Associated Cell Death. Clin. Cancer Res..

[168] Ha E.J., Kim K.Y., Kim C.E., Jun D.Y., Kim Y.H. (2019). Enhancement of Quercetin-Induced Apoptosis by Cotreatment with Autophagy Inhibitor Is Associated with Augmentation of BAK-Dependent Mitochondrial Pathway in Jurkat T Cells. Oxidative Med. Cell. Longev..

[169] Zhao K., Yu M., Zhu Y., Liu D., Wu Q., Hu Y. (2017). EGR-1/ASPP1 inter-regulatory loop promotes apoptosis by inhibiting cyto-protective autophagy. Cell Death Dis..

[170] Vigneron A.M., Ludwig R.L., Vousden K.H. (2010). Cytoplasmic ASPP1 inhibits apoptosis through the control of YAP. Genes Dev..

[171] Granato M., Rizzello C., Romeo M.A., Yadav S., Santarelli R., D’Orazi G., Faggioni A., Cirone M. (2016). Concomitant reduction of c-Myc expression and PI3K/AKT/mTOR signaling by quercetin induces a strong cytotoxic effect against Burkitt’s lymphoma. Int. J. Biochem. Cell Biol..

[172] Du F., Feng Y., Fang J., Yang M. (2015). MicroRNA-143 enhances chemosensitivity of Quercetin through autophagy inhibition via target GABARAPL1 in gastric cancer cells. Biomed. Pharmacother..

[173] Cheung C.W., Gibbons N., Johnson D.W., Nicol D.L. (2010). Silibinin—A Promising New Treatment for Cancer. Anti-Cancer Agents Med. Chem..

[174] Bai Z.-L., Tay V., Guo S.-Z., Ren J., Shu M.-G. (2018). Silibinin Induced Human Glioblastoma Cell Apoptosis Concomitant with Autophagy through Simultaneous Inhibition of mTOR and YAP. BioMed Res. Int..

[175] Wang C., He C., Lu S., Wang X., Wang L., Liang S., Wang X., Piao M., Cui J., Chi G. (2020). Autophagy activated by silibinin contributes to glioma cell death via induction of oxidative stress-mediated BNIP3-dependent nuclear translocation of AIF. Cell Death Dis..

[176] Jiang K., Wang W., Jin X., Wang Z., Ji Z., Meng G. (2015). Silibinin, a natural flavonoid, induces autophagy via ROS-dependent mitochondrial dysfunction and loss of ATP involving BNIP3 in human MCF7 breast cancer cells. Oncol. Rep..

[177] Liu W., Ji Y., Sun Y., Si L., Fu J., Hayashi T., Onodera S., Ikejima T. (2020). Estrogen receptors participate in silibinin-caused nuclear translocation of apoptosis-inducing factor in human breast cancer MCF-7 cells. Arch. Biochem. Biophys..

[178] Si L., Fu J., Liu W., Hayashi T., Nie Y., Mizuno K., Hattori S., Fujisaki H., Onodera S., Ikejima T. (2020). Silibinin inhibits migration and invasion of breast cancer MDA-MB-231 cells through induction of mitochondrial fusion. Mol. Cell. Biochem..

[179] Si L., Fu J., Liu W., Hayashi T., Mizuno K., Hattori S., Fujisaki H., Onodera S., Ikejima T. (2020). Silibinin-induced mitochondria fission leads to mitophagy, which attenuates silibinin-induced apoptosis in MCF-7 and MDA-MB-231 cells. Arch. Biochem. Biophys..

[180] Zheng N., Liu L., Liu W.-W., Li F., Hayashi T., Tashiro S.-I., Onodera S., Ikejima T. (2017). Crosstalk of ROS/RNS and autophagy in silibinin-induced apoptosis of MCF-7 human breast cancer cells in vitro. Acta Pharmacol. Sin..

[181] Zheng N., Zhang P., Huang H., Liu W., Hayashi T., Zang L., Zhang Y., Liu L., Xia M., Tashiro S. (2015). ERα down-regulation plays a key role in silibinin-induced autophagy and apoptosis in human breast cancer MCF-7 cells. J. Pharmacol. Sci..

[182] Fan S., Qi M., Yü Y., Li L., Yao G., Tashiro S., Onodera S., Ikejima T. (2012). P53 activation plays a crucial role in silibinin induced ROS generation via PUMA and JNK. Free Radic. Res..

[183] Zhang X., Jiang J., Chen Z., Cao M. (2019). Silibinin inhibited autophagy and mitochondrial apoptosis in pancreatic carcinoma by activating JNK/SAPK signaling. Pathol. Res. Pract..

[184] Li F., Ma Z., Guan Z., Chen Y., Wu K., Guo P., Wang X., He D., Zeng J. (2015). Autophagy Induction by Silibinin Positively Contributes to Its Anti-Metastatic Capacity via AMPK/mTOR Pathway in Renal Cell Carcinoma. Int. J. Mol. Sci..

[185] Fan Y., Hou T., Dan W., Liu T., Luan J., Liu B., Li L., Zeng J. (2020). Silibinin inhibits epithelial-mesenchymal transition of renal cell carcinoma through autophagy-dependent Wnt/β-catenin signaling. Int. J. Mol. Med..

[186] Kauntz H., Bousserouel S., Gossé F., Raul F. (2011). Silibinin triggers apoptotic signaling pathways and autophagic survival response in human colon adenocarcinoma cells and their derived metastatic cells. Apoptosis.

[187] Duan W.-J., Li Q.-S., Xia M.-Y., Tashiro S., Onodera S., Ikejima T. (2011). Silibinin activated ROS–p38–NF-κB positive feedback and induced autophagic death in human fibrosarcoma HT1080 cells. J. Asian Nat. Prod. Res..

[188] Liu W., Otkur W., Li L., Wang Q., He H., Ye Y., Zhang Y., Hayashi T., Tashiro S., Onodera S. (2013). Autophagy induced by silibinin protects human epidermoid carcinoma A431 cells from UVB-induced apoptosis. J. Photochem. Photobiol. B Biol..

[189] Wang Q., Liu W., Zeng H., Xie X., Zang G., Ye Y., Tashiro S., Onodera S., Jiang S., Ikejima T. (2013). p53-mediated autophagy adjustment is involved in the protection of silibinin against murine dermal inflammation and epidermal apoptosis induced by UVB irradiation. J. Asian Nat. Prod. Res..

[190] Mohan A., Narayanan S., Sethuraman S., Krishnan U.M. (2013). Combinations of plant polyphenols & anti-cancer molecules: A novel treatment strategy for cancer chemotherapy. Anti-Cancer Agents Med. Chem..

[191] Forbes J.M., Cooper M.E. (2013). Mechanisms of Diabetic Complications. Physiol. Rev..

[192] Silveira A.C., Dias J.P., Santos V.M., Oliveira P.F., Alves M.G., Rato L., Silva B.M. (2019). The Action of Polyphenols in Diabetes Mellitus and Alzheimer’s Disease: A Common Agent for Overlapping Pathologies. Curr. Neuropharmacol..

[193] Asmat U., Abad K., Ismail K. (2016). Diabetes mellitus and oxidative stress—A concise review. Saudi Pharm. J..

[194] Ma X., Chen Z., Wang L., Wang G., Wang Z., Dong X., Wen B., Zhang Z. (2018). The Pathogenesis of Diabetes Mellitus by Oxidative Stress and Inflammation: Its Inhibition by Berberine. Front. Pharmacol..

[195] Morón E.B., Abad-Jiménez Z., De Marañón A.M., Iannantuoni F., López E., López-Domènech S., Salom C., Jover A., Mora V., Roldan I. (2019). Relationship Between Oxidative Stress, ER Stress, and Inflammation in Type 2 Diabetes: The Battle Continues. J. Clin. Med..

[196] Yorimitsu T., Klionsky D.J. (2005). Autophagy: Molecular machinery for self-eating. Cell Death Differ..

[197] Singh R., Kaushik S., Wang Y., Xiang Y., Novak I., Komatsu M., Tanaka K., Cuervo A.M., Czaja M.J. (2009). Autophagy regulates lipid metabolism. Nature.

[198] Liu H.-Y., Han J., Cao S.Y., Hong T., Zhuo D., Shi J., Liu Z., Cao W. (2009). Hepatic Autophagy Is Suppressed in the Presence of Insulin Resistance and Hyperinsulinemia: Inhibition of FoxO1-dependent expression of key autophagy genes by insulin. J. Biol. Chem..

[199] Ebato C., Uchida T., Arakawa M., Komatsu M., Ueno T., Komiya K., Azuma K., Hirose T., Tanaka K., Kominami E. (2008). Autophagy Is Important in Islet Homeostasis and Compensatory Increase of Beta Cell Mass in Response to High-Fat Diet. Cell Metab..

[200] Fujitani Y., Kawamori R., Watada H. (2009). The role of autophagy in pancreatic β-cell and diabetes. Autophagy.

[201] Masini M., Bugliani M., Lupi R., Del Guerra S., Boggi U., Filipponi F., Marselli L., Masiello P., Marchetti P. (2009). Autophagy in human type 2 diabetes pancreatic beta cells. Diabetologia.

[202] Scalbert A., Manach C., Morand C., Rémésy C., Jiménez L. (2005). Dietary Polyphenols and the Prevention of Diseases. Crit. Rev. Food Sci. Nutr..

[203] Guasch-Ferré M., Merino J., Sun Q., Fitó M., Salas-Salvadó J. (2017). Dietary Polyphenols, Mediterranean Diet, Prediabetes, and Type 2 Diabetes: A Narrative Review of the Evidence. Oxidative Med. Cell. Longev..

[204] Lv W., Zhang J., Jiao A., Wang B., Chen B., Lin J. (2019). Resveratrol attenuates hIAPP amyloid formation and restores the insulin secretion ability in hIAPP-INS1 cell line via enhancing autophagy. Can. J. Physiol. Pharmacol..

[205] Wang D., Sun H., Song G., Yang Y., Zou X., Han P., Li S. (2018). Resveratrol Improves Muscle Atrophy by Modulating Mitochondrial Quality Control in STZ-Induced Diabetic Mice. Mol. Nutr. Food Res..

[206] Darwish M.A., Abdel-Bakky M.S., Messiha B.A.S., Abo-Saif A.A., Abo-Youssef A.M. (2021). Resveratrol mitigates pancreatic TF activation and autophagy-mediated beta cell death via inhibition of CXCL16/ox-LDL pathway: A novel protective mechanism against type 1 diabetes mellitus in mice. Eur. J. Pharmacol..

[207] Xu K., Liu X.-F., Ke Z.-Q., Yao Q., Guo S., Liu C. (2018). Resveratrol Modulates Apoptosis and Autophagy Induced by High Glucose and Palmitate in Cardiac Cells. Cell. Physiol. Biochem..

[208] Wang G.-Y., Bi Y.-G., Liu X.-D., Han J.-F., Wei M., Zhang Q.-Y. (2017). Upregulation of connexin 43 and apoptosis-associated protein expression by high glucose in H9c2 cells was improved by resveratrol via the autophagy signaling pathway. Mol. Med. Rep..

[209] Ji J., Zhao Y., Na C., Yang M., Zhu X., Shi H., Gan W., Zhang A. (2019). Connexin 43-autophagy loop in the podocyte injury of diabetic nephropathy. Int. J. Mol. Med..

[210] Wang B., Yang Q., Sun Y.Y., Xing Y.F., Wang Y.B., Lu X.T., Bai W.W., Liu X.Q., Zhao Y.X. (2014). Resveratrol-enhanced autophagic flux ameliorates myocardial oxidative stress injury in diabetic mice. J. Cell. Mol. Med..

[211] Kuchitsu Y., Fukuda M. (2018). Revisiting Rab7 Functions in Mammalian Autophagy: Rab7 Knockout Studies. Cells.

[212] Qu X., Chen X., Shi Q., Wang X., Wang D., Yang L. (2019). Resveratrol alleviates ischemia/reperfusion injury of diabetic myocardium via inducing autophagy. Exp. Ther. Med..

[213] Huang S.-S., Ding D.-F., Chen S., Dong C.-L., Ye X.-L., Yuan Y.-G., Feng Y.-M., You N., Xu J.-R., Miao H. (2017). Resveratrol protects podocytes against apoptosis via stimulation of autophagy in a mouse model of diabetic nephropathy. Sci. Rep..

[214] Ma L., Fu R., Duan Z., Lu J., Gao J., Tian L., Lv Z., Chen Z., Han J., Jia L. (2016). Sirt1 is essential for resveratrol enhancement of hypoxia-induced autophagy in the type 2 diabetic nephropathy rat. Pathol. Res. Pract..

[215] Xu X.H., Ding D.F., Yong H.J., Dong C.L., You N., Ye X.L., Pan M.L., Ma J.H., You Q., Lu Y.B. (2017). Resveratrol tran-scriptionally regulates miRNA-18a-5p expression ameliorating diabetic nephropathy via increasing autophagy. Eur. Rev. Med. Pharmacol. Sci..

[216] Zhao Y.-H., Fan Y.J. (2020). Resveratrol improves lipid metabolism in diabetic nephropathy rats. Front. Biosci..

[217] Hu Q., Mao Y., Liu M., Luo R., Jiang R., Guo F. (2020). The active nuclear form of SREBP1 amplifies ER stress and autophagy via regulation of PERK. FEBS J..

[218] Zhao Y., Song W., Wang Z., Wang Z., Jin X., Xu J., Bai L., Li Y., Cui J., Cai L. (2018). Resveratrol attenuates testicular apoptosis in type 1 diabetic mice: Role of Akt-mediated Nrf2 activation and p62-dependent Keap1 degradation. Redox Biol..

[219] Wang L., Sun X., Zhu M., Du J., Xu J., Qin X., Xu X., Song E. (2019). Epigallocatechin-3-gallate stimulates autophagy and reduces apoptosis levels in retinal Müller cells under high-glucose conditions. Exp. Cell Res..

[220] Yan J., Feng Z., Liu J., Shen W., Wang Y., Wertz K., Weber P., Long J., Liu J. (2012). Enhanced autophagy plays a cardinal role in mitochondrial dysfunction in type 2 diabetic Goto–Kakizaki (GK) rats: Ameliorating effects of (−)-epigallocatechin-3-gallate. J. Nutr. Biochem..

[221] Liu J., Tang Y., Feng Z., Long J. (2014). (–)-Epigallocatechin-3-gallate attenuated myocardial mitochondrial dysfunction and autophagy in diabetic Goto–Kakizaki rats. Free Radic. Res..

[222] Assimacopoulos-Jeannet F. (2004). Fat storage in pancreas and in insulin-sensitive tissues in pathogenesis of type 2 diabetes. Int. J. Obes. Relat. Metab. Disord..

[223] Varshney R., Gupta S., Roy P. (2017). Cytoprotective effect of kaempferol against palmitic acid-induced pancreatic β-cell death through modulation of autophagy via AMPK/mTOR signaling pathway. Mol. Cell. Endocrinol..

[224] Varshney R., Varshney R., Mishra R., Gupta S., Sircar D., Roy P. (2018). Kaempferol alleviates palmitic acid-induced lipid stores, endoplasmic reticulum stress and pancreatic β-cell dysfunction through AMPK/mTOR-mediated lipophagy. J. Nutr. Biochem..

[225] Yang J., Sun Y., Xu F., Liu W., Hayashi T., Onodera S., Tashiro S.-I., Ikejima T. (2018). Involvement of estrogen receptors in silibinin protection of pancreatic β-cells from TNFα- or IL-1β-induced cytotoxicity. Biomed. Pharmacother..

[226] Rezabakhsh A., Fathi F., Bagheri H.S., Malekinejad H., Montaseri A., Rahbarghazi R., Garjani A. (2018). Silibinin protects human endothelial cells from high glucose-induced injury by enhancing autophagic response. J. Cell. Biochem..

[227] Wang Q., Liu M., Liu W.-W., Hao W.-B., Tashiro S., Onodera S., Ikejima T. (2012). In vivorecovery effect of silibinin treatment on streptozotocin-induced diabetic mice is associated with the modulations of sirt-1 expression and autophagy in pancreatic β-cell. J. Asian Nat. Prod. Res..

[228] Matboli M., Ibrahim D., Hasanin A.H., Hassan M.K., Habib E.K., Bekhet M.M., Afifi A.M., Eissa S. (2021). Epigenetic modulation of autophagy genes linked to diabetic nephropathy by administration of isorhamnetin in Type 2 diabetes mellitus rats. Epigenomics.

[229] Matboli M., Saad M., Hasanin A.H., Saleh L.A., Baher W., Bekhet M.M., Eissa S. (2021). New insight into the role of isorhamnetin as a regulator of insulin signaling pathway in type 2 diabetes mellitus rat model: Molecular and computational approach. Biomed. Pharmacother..

[230] Wang G., Liang J., Gao L.-R., Si Z.-P., Zhang X.-T., Liang G., Yan Y., Li K., Cheng X., Bao Y. (2018). Baicalin administration attenuates hyperglycemia-induced malformation of cardiovascular system. Cell Death Dis..

[231] Zhang Y., Cao Y., Chen J., Qin H., Yang L. (2019). A New Possible Mechanism by Which Punicalagin Protects against Liver Injury Induced by Type 2 Diabetes Mellitus: Upregulation of Autophagy via the Akt/FoxO3a Signaling Pathway. J. Agric. Food Chem..

[232] Yin L., Chen X., Li N., Jia W., Wang N., Hou B., Yang H., Zhang L., Qiang G., Yang X. (2021). Puerarin ameliorates skeletal muscle wasting and fiber type transformation in STZ-induced type 1 diabetic rats. Biomed. Pharmacother..

[233] Wang X.-Y., Zhu B.-R., Jia Q., Li Y.-M., Wang T., Wang H.-Y. (2020). Cinnamtannin D1 Protects Pancreatic β-Cells from Glucolipotoxicity-Induced Apoptosis by Enhancement of Autophagy In Vitro and In Vivo. J. Agric. Food Chem..

[234] Guo L., Tan K., Luo Q., Bai X. (2020). Dihydromyricetin promotes autophagy and attenuates renal interstitial fibrosis by regulating miR-155-5p/PTEN signaling in diabetic nephropathy. Bosn. J. Basic Med. Sci..

[235] Ni T., Lin N., Lu W., Sun Z., Lin H., Chi J., Guo H. (2020). Dihydromyricetin Prevents Diabetic Cardiomyopathy via miR-34a Suppression by Activating Autophagy. Cardiovasc. Drugs Ther..

[236] Zhang J., Li A.-M., Liu B.-X., Han F., Liu F., Sun S.-P., Li X., Cui S.-J., Xian S.-Z., Kong G.-Q. (2013). Effect of icarisid II on diabetic rats with erectile dysfunction and its potential mechanism via assessment of AGEs, autophagy, mTOR and the NO–cGMP pathway. Asian J. Androl..

[237] Zhang J., Li S., Zhang S., Wang Y., Jin S., Zhao C., Yang W., Liu Y., Kong G. (2020). Effect of Icariside II and Metformin on Penile Erectile Function, Histological Structure, Mitochondrial Autophagy, Glucose-Lipid Metabolism, Angiotensin II and Sex Hormone in Type 2 Diabetic Rats with Erectile Dysfunction. Sex. Med..

[238] Wang K., Peng S., Xiong S., Niu A., Xia M., Xiong X., Zeng G., Huang Q. (2020). Naringin inhibits autophagy mediated by PI3K-Akt-mTOR pathway to ameliorate endothelial cell dysfunction induced by high glucose/high fat stress. Eur. J. Pharmacol..

[239] Lai D., Huang M., Zhao L., Tian Y., Li Y., Liu D., Wu Y., Deng F. (2019). Delphinidin-induced autophagy protects pancreatic β cells against apoptosis resulting from high-glucose stress via AMPK signaling pathway. Acta Biochim. Biophys. Sin..

[240] Su H., Xie L., Xu Y., Ke H., Bao T., Li Y., Chen W. (2020). Pelargonidin-3-O-glucoside Derived from Wild Raspberry Exerts Antihyperglycemic Effect by Inducing Autophagy and Modulating Gut Microbiota. J. Agric. Food Chem..

[241] Wang X., Wang W., Li L., Perry G., Lee H.-G., Zhu X. (2014). Oxidative stress and mitochondrial dysfunction in Alzheimer’s disease. Biochim. Biophys. Acta (BBA) Mol. Basis Dis..

[242] Behl C. (2005). Oxidative stress in Alzheimer’s Disease: Implications for Prevention and Therapy. Subcell. Biochem..

[243] Andersen J.K. (2004). Oxidative stress in neurodegeneration: Cause or consequence?. Nat. Med..

[244] Behl C., Trapp T., Skutella T., Holsboer F. (1997). Protection against oxidative stress-induced neuronal cell death—A novel role for RU486. Eur. J. Neurosci..

[245] Elfawy H.A., Das B. (2018). Crosstalk between mitochondrial dysfunction, oxidative stress, and age related neurodegenerative disease: Etiologies and therapeutic strategies. Life Sci..

[246] Hooper N.M. (2005). Roles of proteolysis and lipid rafts in the processing of the amyloid precursor protein and prion protein. Biochem. Soc. Trans..

[247] Ananbeh H., Vodicka P., Skalnikova H. (2021). Emerging Roles of Exosomes in Huntington’s Disease. Int. J. Mol. Sci..

[248] Bisi N., Feni L., Peqini K., Pérez-Peña H., Ongeri S., Pieraccini S., Pellegrino S. (2021). α-Synuclein: An All-Inclusive Trip Around its Structure, Influencing Factors and Applied Techniques. Front. Chem..

[249] Meccariello R., D’Angelo S. (2021). Impact of Polyphenolic-Food on Longevity: An Elixir of Life. An Overview. Antioxidants.

[250] Van Hung P. (2016). Phenolic Compounds of Cereals and Their Antioxidant Capacity. Crit. Rev. Food Sci. Nutr..

[251] Grosso G., Estruch R. (2016). Nut consumption and age-related disease. Maturitas.

[252] Szwajgier D., Borowiec K., Pustelniak K. (2017). The Neuroprotective Effects of Phenolic Acids: Molecular Mechanism of Action. Nutrients.

[253] Li G., Ruan L., Chen R., Wang R., Xie X., Zhang M., Chen L., Yan Q., Reed M., Chen J. (2015). Synergistic antidepressant-like effect of ferulic acid in combination with piperine: Involvement of monoaminergic system. Metab. Brain Dis..

[254] Chen J., Lin D., Zhang C., Li G., Zhang N., Ruan L., Yan Q., Li J., Yu X., Xie X. (2015). Antidepressant-like effects of ferulic acid: Involvement of serotonergic and norepinergic systems. Metab. Brain Dis..

[255] Lenzi J., Rodrigues A.F., Rós A.D.S., De Castro B.B., De Lima D.D., Magro D.D.D., Zeni A.L.B. (2015). Ferulic acid chronic treatment exerts antidepressant-like effect: Role of antioxidant defense system. Metab. Brain Dis..

[256] Liu Y.-M., Hu C.-Y., Shen J.-D., Wu S.-H., Li Y.-C., Yi L.-T. (2017). Elevation of synaptic protein is associated with the antidepressant-like effects of ferulic acid in a chronic model of depression. Physiol. Behav..

[257] Aswar M., Patil V. (2016). Ferulic acid ameliorates chronic constriction injury induced painful neuropathy in rats. Inflammopharmacology.

[258] Nagarajan S., Chellappan D.R., Chinnaswamy P., Thulasingam S. (2015). Ferulic acid pretreatment mitigates MPTP-induced motor impairment and histopathological alterations in C57BL/6 mice. Pharm. Biol..

[259] Fazili N.A., Naeem A. (2015). Anti-fibrillation potency of caffeic acid against an antidepressant induced fibrillogenesis of human α-synuclein: Implications for Parkinson’s disease. Biochimie.

[260] Bak J., Kim H.J., Kim S.Y., Choi Y.-S. (2016). Neuroprotective effect of caffeic acid phenethyl ester in 3-nitropropionic acid-induced striatal neurotoxicity. Korean J. Physiol. Pharmacol..

[261] Zhang Y., Wu Q., Zhang L., Wang Q., Yang Z., Liu J., Feng L. (2019). Caffeic acid reduces A53T α-synuclein by activating JNK/Bcl-2-mediated autophagy in vitro and improves behaviour and protects dopaminergic neurons in a mouse model of Parkinson’s disease. Pharmacol. Res..

[262] Tomiyama R., Takakura K., Takatou S., Le T.M., Nishiuchi T., Nakamura Y., Konishi T., Matsugo S., Hori O. (2018). 3,4-dihydroxybenzalacetone and caffeic acid phenethyl ester induce preconditioning ER stress and autophagy in SH-SY5Y cells. J. Cell. Physiol..

[263] Kim H.M., Kim Y., Lee E.S., Huh J.H., Chung C.H. (2018). Caffeic acid ameliorates hepatic steatosis and reduces ER stress in high fat diet–induced obese mice by regulating autophagy. Nutrition.

[264] Wu J., Chen H., Li H., Tang Y., Yang L., Cao S., Qin D. (2016). Antidepressant Potential of Chlorogenic Acid-Enriched Extract from Eucommia ulmoides Oliver Bark with Neuron Protection and Promotion of Serotonin Release through Enhancing Synapsin I Expression. Molecules.

[265] Aseervatham G.S., Suryakala U., Sundaram S., Bose P.C., Sivasudha T. (2016). Expression pattern of NMDA receptors reveals antiepileptic potential of apigenin 8-C-glucoside and chlorogenic acid in pilocarpine induced epileptic mice. Biomed. Pharmacother..

[266] Loos B., Engelbrecht A.-M., Lockshin R.A., Klionsky D.J., Zakeri Z. (2013). The variability of autophagy and cell death susceptibility. Autophagy.

[267] Gao L., Li X., Meng S., Ma T., Wan L., Xu S. (2020). Chlorogenic Acid Alleviates Aβ_25-35_-Induced Autophagy and Cognitive Impairment via the mTOR/TFEB Signaling Pathway. Drug Des. Dev. Ther..

[268] Coelho V.R., Vieira C.G., de Souza L.P., Moysés F., Basso C., Papke D.K., Pires T.R., Siqueira I.R., Picada J.N., Pereira P. (2015). Antiepileptogenic, antioxidant and genotoxic evaluation of rosmarinic acid and its metabolite caffeic acid in mice. Life Sci..

[269] Khamse S., Sadr S.S., Roghani M., Hasanzadeh G., Mohammadian M. (2015). Rosmarinic acid exerts a neuroprotective effect in the kainate rat model of temporal lobe epilepsy: Underlying mechanisms. Pharm. Biol..

[270] Kondo S., El Omri A., Han J., Isoda H. (2015). Antidepressant-like effects of rosmarinic acid through mitogen-activated protein kinase phosphatase-1 and brain-derived neurotrophic factor modulation. J. Funct. Foods.

[271] Shan Y., Wang D.-D., Xu Y.-X., Wang C., Cao L., Liu Y.-S., Zhu C.-Q. (2016). Aging as a Precipitating Factor in Chronic Restraint Stress-Induced Tau Aggregation Pathology, and the Protective Effects of Rosmarinic Acid. J. Alzheimer’s Dis..

[272] Yao Y., Huang H.-Y., Yang Y.-X., Guo J.-Y. (2015). Cinnamic aldehyde treatment alleviates chronic unexpected stress-induced depressive-like behaviors via targeting cyclooxygenase-2 in mid-aged rats. J. Ethnopharmacol..

[273] Yin X., Su X., Wang X., Su J., Lian Y., Zhang X. (2015). Effects of protocatechuic acid on expression of D2DR, iNOS, and TH in striatum and midbrain of Parkinson’s disease model mice. Chin. Tradit. Herb. Drugs.

[274] Cammisuli D.M., Cammisuli S.M., Fusi J., Franzoni F., Pruneti C. (2019). Parkinson’s Disease–Mild Cognitive Impairment (PD-MCI): A Useful Summary of Update Knowledge. Front. Aging Neurosci..

[275] Havsteen B. (1983). Flavonoids, a class of natural products of high pharmacological potency. Biochem. Pharmacol..

[276] Cushnie T., Lamb A.J. (2005). Antimicrobial activity of flavonoids. Int. J. Antimicrob. Agents.

[277] Prasain J.K., Wang C.-C., Barnes S. (2004). Mass spectrometric methods for the determination of flavonoids in biological samples. Free Radic. Biol. Med..

[278] Socci V., Tempesta D., Desideri G., De Gennaro L., Ferrara M. (2017). Enhancing Human Cognition with Cocoa Flavonoids. Front. Nutr..

[279] Bakoyiannis I., Daskalopoulou A., Pergialiotis V., Perrea D. (2019). Phytochemicals and cognitive health: Are flavonoids doing the trick?. Biomed. Pharmacother..

[280] Mastroiacovo D., Kwik-Uribe C., Grassi D., Necozione S., Raffaele A., Pistacchio L., Righetti R., Bocale R., Lechiara M.C., Marini C. (2015). Cocoa flavanol consumption improves cognitive function, blood pressure control, and metabolic profile in elderly subjects: The Cocoa, Cognition, and Aging (CoCoA) Study—a randomized controlled trial. Am. J. Clin. Nutr..

[281] Schroeter H., Boyd C., Spencer J.P., Williams R.J., Cadenas E., Rice-Evans C. (2002). MAPK signaling in neurodegeneration: Influences of flavonoids and of nitric oxide. Neurobiol. Aging.

[282] Spencer J.P. (2008). Flavonoids: Modulators of brain function?. Br. J. Nutr..

[283] Spencer J.P., Vauzour D., Rendeiro C. (2009). Flavonoids and cognition: The molecular mechanisms underlying their behavioural effects. Arch. Biochem. Biophys..

[284] Williams R.J., Spencer J.P.E. (2012). Flavonoids, cognition, and dementia: Actions, mechanisms, and potential therapeutic utility for Alzheimer disease. Free Radic. Biol. Med..

[285] Esatbeyoglu T., Ewald P., Yasui Y., Yokokawa H., Wagner A.E., Matsugo S., Winterhalter P., Rimbach G. (2016). Chemical Characterization, Free Radical Scavenging, and Cellular Antioxidant and Anti-Inflammatory Properties of a Stilbenoid-Rich Root Extract ofVitis vinifera. Oxidative Med. Cell. Longev..

[286] Akinwumi B.C., Bordun K.-A.M., Anderson H.D. (2018). Biological Activities of Stilbenoids. Int. J. Mol. Sci..

[287] Wen H., Fu Z., Wei Y., Zhang X., Ma L., Gu L., Li J. (2018). Antioxidant Activity and Neuroprotective Activity of Stilbenoids in Rat Primary Cortex Neurons via the PI3K/Akt Signalling Pathway. Molecules.

[288] Simonian N.A., Coyle J.T. (1996). Oxidative Stress in Neurodegenerative Diseases. Annu. Rev. Pharmacol. Toxicol..

[289] Lu K.-T., Ko M.-C., Chen B.-Y., Huang J.-C., Hsieh C.-W., Lee M.-C., Chiou R.Y.Y., Wung B.-S., Peng C.-H., Yang Y.-L. (2008). Neuroprotective Effects of Resveratrol on MPTP-Induced Neuron Loss Mediated by Free Radical Scavenging. J. Agric. Food Chem..

[290] Saleh M.C., Connell B.J., Saleh T.M. (2013). Resveratrol induced neuroprotection is mediated via both estrogen receptor subtypes, ERα and ERβ. Neurosci. Lett..

[291] Sadigh-Eteghad S., Sabermarouf B., Majdi A., Talebi M., Farhoudi M., Mahmoudi J. (2015). Amyloid-Beta: A Crucial Factor in Alzheimer’s Disease. Med. Princ. Pract..

[292] Butterfield D.A., Castegna A., Lauderback C.M., Drake J. (2002). Evidence that amyloid beta-peptide-induced lipid peroxidation and its sequelae in Alzheimer’s disease brain contribute to neuronal death1. Neurobiol. Aging.

[293] Marambaud P., Zhao H., Davies P. (2005). Resveratrol Promotes Clearance of Alzheimer’s Disease Amyloid-β Peptides. J. Biol. Chem..

[294] Yang H., Zhang A., Zhang Y., Ma S., Wang C. (2016). Resveratrol Pretreatment Protected against Cerebral Ischemia/Reperfusion Injury in Rats via Expansion of T Regulatory Cells. J. Stroke Cerebrovasc. Dis..

[295] Ren J., Fan C., Chen N., Huang J., Yang Q. (2011). Resveratrol Pretreatment Attenuates Cerebral Ischemic Injury by Upregulating Expression of Transcription Factor Nrf2 and HO-1 in Rats. Neurochem. Res..

[296] Hou Y., Wang K., Wan W., Cheng Y., Pu X., Ye X. (2018). Resveratrol provides neuroprotection by regulating the JAK2/STAT3/PI3K/AKT/mTOR pathway after stroke in rats. Genes Dis..

